# Antifungal activity of volatile and non-volatile metabolites of endophytes of *Chloranthus elatior* Sw.

**DOI:** 10.3389/fpls.2023.1156323

**Published:** 2023-05-17

**Authors:** Hiran Kanti Santra, Debdulal Banerjee

**Affiliations:** ^1^ Microbiology and Microbial Biotechnology Laboratory, Department of Botany and Forestry, Vidyasagar University, Midnapore, India; ^2^ Center for Life Sciences, Vidyasagar University, Midnapore, India

**Keywords:** endophytic fungi, VOCs, *M. fructicola*, cherry fruit, mycofumigation

## Abstract

Agriculture crops that have fungal infections suffer significant economic losses and reduced crop output. Chemical fungicides are used to tackle the problem, although this has additional detrimental side effects. There is an urgent need for safe and novel antifungals. Volatiles from plant-beneficial endophytic fungi are considered promising alternatives for the biological control of fungal pathogens as a sustainable approach in an agroecosystem. In the present investigation, a volatile-emitting sterile endophytic fungus, *Diaporthe* sp. CEL3 with bio-fumigation activity, was isolated from leaves of the ethnomedicinal plant *Chloranthus elatior* Sw., collected from the Passighat forest of North-East India. The camphor odor volatiles of CEL3 showed an inhibitory effect against eight fungal pathogens *in vitro* and minimized the infections of *Monilinia fructicola*, a causal agent of cherry fruit rot, in VOC-exposed cherry fruits. *Rhizoctonia solani*, *Botrytis cinerea*, *Pythium ultimum*, and *M*. *fructicola* were maximally inhibited up to 51.5%, 55.8%, 61.9%, and 78.5%, respectively, in comparison to control by the volatiles. Another isolate, CEL7, identified as *Curvularia* sp., synthesized non-volatile, soluble antifungal metabolites in its cell-free extracts and exhibited antifungal action. Bioassay-guided fractionation revealed the presence of imidazole compounds- (2-aminoethyl)-1H-imidazole-2-carbaldehyde, Pyrazole 4, 5 imidazole, 1-formyl 3-ethyl, phenol compounds-Phenol, 4-[2-(methylamino) ethyl]-, 6-Nitro-3-chlorophenol, Phenol, 2,4,6-tri-tert-butyl-, etc., in the cell-free extracts, with a MIC value of 250–2,000 µg ml^−1^. Optimum VOC emission was achieved in a modified PDA medium with instantly smashed potato (150 g L^−1^), dextrose (20 g L^−1^), wheat husk (20 g L^−1^), and yeast extract (20 g L^−1^), with additional salts. Interestingly, endophytic CEL3 emitted different types of volatiles, and trans-verbenol (32.25%), geraniol (30.32%), trans-ocimenol (12.90%), and mentha-4,8-diene (5.16%) were the prime ones. These VOCs cause lethal leakage of protein and necessary intracellular molecules from the fungal pathogens. Thus, CEL3 could potentially be used as a bio-fumigating agent to control post-harvest infections caused by fungal pathogens. This study opens a new approach to the use of endophytic fungi in biocontrol.

## Introduction

Endophytes are a unique class of microbial organisms that invade plant tissues without having any negative effects (Petrini, 1986; Hirsch and Braun, 1992). More than one million fungal endophytes are spread among around 300,000 species of terrestrial plants on this planet, according to an approximation (Jia et al., 2016). Metabolites of endophytic fungi and bacteria are unique in terms of structures and functions and are a potent source of bioactive compounds with multidomain utility in the sectors of pharmaceuticals and agrochemicals (Schulz et al., 2002; Deshmukh et al., 2015). Endophytes have co-evolved with their host plants and shared valuable genes together (Stierle et al., 1993). Thus, they can produce compounds with similar activities to their hosts (Tan and Zou, 2001). Production of the anticancer compound taxol from both the host plant *Taxus brevifolia* and its endophytic fungal isolate *Taxomyces andreanae* is a classic example of that fact (Stierle et al., 1993; Xu et al., 2008).

Endophyte-synthesized metabolites are primarily of two types: volatile and non-volatile (Mitchell et al., 2009). Volatile metabolites are considered volatile organic compounds (VOCs) and are sometimes scent-bearing and mostly antibiotic in nature (Strobel et al., 2001). Previous reports have elucidated that VOCs of endophytic fungi are vigorously bioactive in nature and are potent microbial biocontrol agents of soil and storage pests, postharvest or storage fungal pathogens, and deadly molds (Mercier and Jimenez, 2007; Park et al., 2010; Pena et al., 2019; Santra and Banerjee, 2022c; Santra and Banerjee, 2022d). It has been observed that a few *Muscodor* species, including *M*. *albus* Worapong, *M*. *sutura* Kudalkar & Sears, *M*. *crispans* Mitchell & Ezra, *M*. *strobeli* Meshram & Kapoor, *M*. *musae* Suwannar. & Lumyong, *M*. *brasiliensis* Pena & Kava, *M*. *darjeelingensis* Meshram & Kapoor, *M*. *indica* Meshram & Saxena, *M*. *tigerii* Saxena & Kapoor, *M*. *camphora* Meshram & Saxena, and *M*. *ghoomensis* Meshram & Saxena, are the potent generator of bioactive VOCs with wide-ranging applications in the field of agriculture. (Strobel et al., 2001; Mercier and Smilanick, 2005; Park et al., 2010; Suwannarach et al., 2013; Mao et al., 2019, Saxena and Strobel, 2021).

Almost every plant studied to date harbors endophytes, but the ethnomedicinal plants, especially those from undisturbed/ecologically sound places, are known to be the most suitable sources of bioactive endophytes (Zaferanloo et al., 2012). That is why Northeast India was selected as a possible spot for the collection of plants in this study. This region harbors a rich biodiversity of endemic species that are popular from an ethnomedicinal point of view among the tribes (Sharma et al., 2021). Being a part of the tropical rain forest of the Indo-Burma Hotspots, Arunachal Pradesh is home to a wide variety of unexplored plant-invading microorganisms (i.e., endophytes) that are crucial for agriculture, nutrient management, and industry (Myers et al., 2000; Bhattacharyya et al., 2011). Tropical rain forests are considered treasures of bioactive endophytes across the globe; be it Brazilian Amazonian rain forests, Central American forests, or Northeastern Indian forests (Strobel et al., 2001; Strobel, 2006; Banerjee et al., 2010a; Banerjee et al., 2014). Endophytic fungi from the flora of Northeast India are reported to hold a variety of bioactivities, including antimicrobial, antioxidative, anti-cancer, and production of extracellular enzymes and plant growth promoters (Gogoi et al., 2008; Bhagobaty and Joshi, 2009; Bhagobaty and Joshi, 2012; Padhi and Tayung, 2013; Banerjee et al., 2014). Reports on endophytes of this genus, *Chloranthus elatior*, are scanty, and only one report documents the root endophyte community; antifungal production has not been evaluated earlier (An et al., 2020). But till now, no report has been made on the endophytes of *C. elatior* Sw. from this geographical spot. So, ours is the first attempt to evaluate the bioactivity of endophytes of this species.

This taxon is mainly distributed in eastern and southern Asia (mainly in tropical and temperate parts) and contains numerous types of terpenes in its metabolome: sesquiterpenoids, dimeric sesquiterpenoids, diterpenoids, and coumarin, which holds lists of uses in Asian folk medicine in the form of antitumor, antifungal, and anti-inflammatory agents (Sun et al., 2012; Wang et al., 2015; Liu et al., 2022). Endophytes of *Chloranthus japonicus* collected from the Qinling Mountains in China are reported to host a rich diversity of broad-spectrum antimicrobial-producing fungi (An et al., 2020).

In the present investigation, volatile metabolites from the endophytic fungus CEL3, an isolate of *C. elatior* Sw. collected from the Passighat forest of Arunachal Pradesh, northeast India, blocked the infection of *Monilinia fructicola* in cherry fruits. Geraniol, trans-ocimenol, and verbenol were the major volatiles. Also, the cell-free metabolites of CEL7, i.e., imidazole and phenol compounds, bactobolin, and actinobolin, inhibit the growth of selected fungal pathogens. Both the cell-free and volatile metabolites are tremendously effective against *M*. *fructicola*, with a MIC and IC_50_ value of 250 µg ml^−1^ and 09.80 µl 50 ml^−1^, respectively. *M*. *fructicola* is responsible for severe losses in important commercial stone fruits such as peach, cherry, nectarine, and almond (Batra, 1991). The degree of *Monilinia* infection depends on critical pre- and post-harvest weather parameters, i.e., humidity percentage, rate of rainfall, and temperature fluctuations (Prusky, 1996; Bonaterra et al., 2003; Mari et al., 2012). Also, post-harvest mishandling of cherries may lead to the wounding of fruits and finally lead to *Monilinia* infection, thus disrupting the worldwide market for cherries as well as other valuable stone fruits. *Monilinia* infection accounts for a loss of 10% of the global stone fruit market (17MT, EPPO, 2012), or 1.7 M/year (Larena et al., 2005; Xu et al., 2007), and even copper-based fungicides become ineffective. This causes over 75% of crop losses on commercial organic farms. So novel and sustainable approaches are in high demand, and volatiles from endophytes are the most promising way to solve the problem of fungal infections and crop loss. Here, we have tried to find bio-based, effective solutions to reduce fungal infections in crops by using endophytic fungi as a biocontrol agent. The study’s goal is to identify a bio-based alternative to potentially reduce or eliminate the usage of dangerous chemical fungicides, which have long-term detrimental effects on human health.

## Materials and methods

### Isolation of endophytic fungi

Healthy and disease-free leaves of *C. elatior* were collected from the hills of Passighat forests (28.07°N, 95.33°E) of Arunachal Pradesh, India, and brought to the Microbiology and Microbial Biotechnology Laboratory, Department of Botany and Forestry, Vidyasagar University, West Bengal, India, during the months of November 2018 and 2019. Fifty leaves from each plant and 400 from eight plants of approximately two to three years of age were collected. They were surface sterilized in 75% ethanol for 1 min, in 0.10% sodium hypochlorite for 10 min, and then, washed with distilled water. Explants were cut into 4 mm × 4 mm pieces and were plated onto water agar (WA) plates and kept IN a Biological Oxygen Demand incubator (SN Solutions, India) shaker at 25°C for 3–5 days (Bills, 1996). Approximately 150-µg ml^−1^ of streptomycin was mixed into the WA to avoid bacterial contamination and bacterial endophytes. The effectiveness of this sterilization and isolation process was cross-checked by the explant imprintation technique described by Schulz et al. (1993). In brief, the aliquots used for explant sterilization were spread on a water–agar medium and incubated under the same conditions to detect the growth of any surface microorganisms. Finally, the fungal hyphae emerging from the explant tissues are transferred to Potato Dextrose Agar (PDA) medium (HiMedia, India) and stocked on PDA slants at 4°C for future use.

### Screening of fungal endophytes for the production of antifungal metabolites

#### *In vitro* antifungal assay of emitted VOCs

A total of twenty-one fungal endophytes were obtained from *C*. *elatior* leaves and were tested for their antifungal VOC emission capability against a variety of fungal pathogens: *Rhizoctonia solani* (MTCC-4634), *Alternaria alternata* (MTCC-3793), *Fusarium oxysporum* (MTCC-284), *Geotrichum candidum* (MTCC-3993), *Botrytis cinerea* (MTCC-8659), *Cercospora beticola* (ATCC-12825), *Ceratocystis ulmi* (ATCC-32437), *Pythium ultimum* (ATCC-200006), *Monilinia fructicola* (ATCC no. 62880) and storage pathogen, *Aspergillus fumigatus* (MTCC-3785) following the methods of Strobel et al. (2001). In brief, a 1-cm-wide strip of agar was cut off from the center of a standard Petri plate (100 mm × 15 mm) of PDA, forming two halves of agar. Endophytic fungi were inoculated on one side of the plate (half-moon agar) and incubated at 24°C for 4–8 days. Test pathogenic fungi were inoculated on the other half-moon agar side of the Petri plate from the 4th until the 8th day. Petri plates were then wrapped two to three times with parafilm and incubated at 24°C for 72–144 h. A plate without a fungal endophyte was used as a control. The colony of fungal pathogens was observed after 6 days of incubation, and inhibition (%) was calculated in comparison to the control plate. The tests were conducted in triplicate. The *M*. *fructicola* pathogen was most effectively inhibited by CEL3 volatiles when compared to other fungal pathogens, and it was subsequently studied *in vivo* in cherry fruits. Additionally, exclusively utilizing this test pathogen, the media optimization for maximum antifungal emission was assessed.

### *In vitro* antifungal activity of the cell-free culture extracts

#### Preparation of cell-free culture extracts

Endophytic fungal hyphal blocks of 0.5 cm × 0.5 cm size were cut off from full-grown PDA plates of isolates. The blocks were inoculated into 100 ml of pre-sterilized and cooled down (temperature of 28°C) Potato Dextrose Broth medium (PDB) in a 250 ml Erlenmeyer flask. Inoculated PDB flasks were incubated at 28°C for 8 to 10 days with mild shaking at 120 rpm in a BOD shaker incubator. Cell-free culture extracts from each broth were prepared by filtering out the cell mass using a Whatman No. 4 filter paper followed by centrifugation at 10,000 rpm for 20 min. The cell-free culture extract is then refrigerated for further studies.

#### Detection of antifungal activity using agar well diffusion technique

The antifungal activity of each cell-free culture extract was checked against pathogenic fungal strains by the agar well diffusion method (Hashem et al., 2022). The agar well diffusion test was performed following the guidelines of the Clinical Laboratory Standard Institute (John, 2008). Pathogens were initially grown n PDA plates and incubated at 24°C for 2–4 days. Cork borers of 5 mm diameter were used to form two holes in the PDA plates, 20 mm from the fungal colony, and cell-free culture extracts (50 µl) of endophytic fungi were poured into the wells. Plates were incubated for 3–5 days at 30°C. After incubation, a clear zone of inhibition was recorded. Fluconazole (1 µg ml^−1^) was used as a standard antifungal drug.

### Identification of the selected isolates and evaluation of diversity indices

Endophytic fungal isolates were identified based on their microscopic morphology. Microscopy structures of the fungal endophytes were taken using a light microscope (Primo Star, Zeiss, Germany). Fungal hyphae and sexual/asexual reproductive bodies were taken onto a grease-free glass slide, stained using 2% cotton blue, and covered with a square coverslip (Bluestar, India). Six isolates were sterile and did not produce any reproductive structures, so they remained unidentified. Out of 21 isolates, the two most potent isolates with maximum antifungal action in terms of volatile and non-volatile antifungal production were sterile, devoid of any reproductive structures, and did not secrete any identifiable pigments. These two isolates were selected for DNA sequencing-based molecular identification following standard procedures. In brief, the organism was identified by an rDNA-based molecular technique because there was no reproductive structure produced by the endophytic fungi even in a medium with carnated leaves. Genomic DNA of the eight-day-old fully grown fungal isolate was obtained (using DNeasy Plant Minikit-Qiagen, Germany), and a polymerase chain reaction was performed using the two universal primers named ITS1 (5’-TCCGTAGGTGAACCTTGCGG-3’) and ITS2 (5’-TCCTCCGCTTATTGATATGC-3’) (Landum et al., 2016). In brief, the 25 µl of reaction volume contained 12.5 µl of 2× PCR Bio Taq Mix Red (PCR Biosystems, UK), 0.4 µM of both the forward and reverse primers, and 10 ng of genomic DNA template. Another set was produced and termed a negative control, where DNA was replaced with distilled water to assure a contamination-free reaction. The PCR (Bio-Rad, USA) program configuration was as follows: 1 min at 95°C, 35 cycles at 95°C for 15 s, then again for 15 s at 55°C, 1 min at 72°C, and, finally, a 10-minute extension at 72°C. Ultimately, the PCR products were separated using 1% agarose gel in 1× TAE buffer (90 mM Tris-acetate and 2 nM EDTA, pH 8.0) and stained with ethidium bromide (0.5 µg ml^−1^), before finally being documented using the BIO-RAD Gel Doc EZ imager version 5.1 (USA). PCR products were sent for direct bi-directional sequencing using the ABI 3730xl Genetic Analyzer (Applied Biosystems, USA) to Bioserve Biotechnologies (India) Pvt. Ltd., A Repro Cell Company, Hyderabad, India. The obtained consensus sequence was used for further study. Sequences were submitted to GenBank and compared to the GenBank database using BLAST. Sequences with higher similarity indices were selected and aligned using the multiple alignment software program Clustal W, and the phylogenetic tree was prepared using MEGA 11 (Tamura et al., 2021). The colonization frequency of each isolate, along with diversity indices like Shannon–Wiener, Simpson’s dominance, Simpson’s diversity, and species richness, was also calculated following standard methods (Simpson, 1951; Hata and Futai, 1995; Gond et al., 2012).

### Solvent extraction of CEL7 metabolites and evaluation of MIC values

Antifungal metabolites synthesized by the endophytic isolate CEL3 were extracted using three different types of organic solvents, i.e., ethyl acetate, n-hexane, and ethyl ether, but ethyl acetate (EA, HiMedia-LR grade) was found to be the most effective extraction solvent in terms of maximum antifungal activity. CEL7 was cultured in 1.5 L of PDB for 8 days at 28°C at 120 rpm in a 2 L bioreactor (Eyela, Japan). Cell-free culture extract was obtained by filtrating the cell mass with a muslin cloth, followed by centrifugation at 10,000 rpm for 20 min. For every 100 ml of the filtrate, 200 ml of ethyl acetate was added and shaken for 30 min using a magnetic stirrer. Lastly, the EA layer was separated using a separating funnel. A vacuum rotary evaporator (Bacca Buchi, Switzerland) was used to evaporate the excess EA under low pressure and temperature (105 millibars, 45°C). Finally, the 250 ml concentrated ethyl acetate were taken and freeze-dried, i.e., lyophilized (Star Scientific Solutions, India). The dried part was weighed and stored for further analysis. The dried part was dissolved in DMSO (dimethyl sulfoxide, HiMedia) for evaluation of antifungal activity following the agar-well diffusion technique. Fluconazole was taken as a positive control. DMSO and EA were used as negative controls. In the case of the MIC assay, first pathogens were individually grown on PDA plates, and after 2–3 days of growth, 50 µl of DMSO with varying concentrations (62.5 µg ml^−1^ to 2 mg ml^−1^) of metabolites were added to the different wells. The clear zone of inhibition was measured and recorded after 2 days. The concentration at which minimum inhibition was detected was reported as the MIC of metabolites against respective pathogens.

### Optimization of fermentation medium of CEL3 for the maximum VOCs emission and antifungal activity

Different types of basal media were evaluated for their effect on the VOC production of CEL3 in terms of scent emission and antifungal activity. The emission of ocimenol, the highest occurrence among other volatiles (discussed later), was assessed qualitatively due to its strong fruity camphor odor. PDA (HIMEDIA, India), PDA medium (instantly smashed potatoes and 2% dextrose), oatmeal agar (OMA, HIMEDIA, India), potato carrot agar (PCA), and malt extract peptone agar (MEPA; malt extract 20 g, peptone 2 g, agar 20 g DW-distilled water, 1 L) were used for the growth and VOC emission of CEL3, and PDA (with instantly smashed potatoes) was found to be the most suitable medium. Along with this basal medium, additional nitrogen sources (yeast extract, peptone, tryptone, NH_4_NO_3_, and urea, 2 g L^−1^) were checked, and yeast extract was found to be the most influential one. Also, the other six ingredients (common cheap waste products), i.e., bagasse, rice hulls, soybean meals, sesame meals, tea seed pomace, and wheat husk, were added individually, and the VOC production was evaluated by both olfactory testing and inhibition of the fungal pathogen’s growth. Each ingredient (3 g) was put into glass beakers and sterilized by autoclaving at 121°C for 20 min. Then, 2% of these ingredients were added to individual PDAs (instantly smashed potatoes and 2% agar), and CEL3 was inoculated into them upon solidification. In each case, the salt concentration of the final selected media was maintained according to the modified M1-D medium (Pinkerton and Strobel, 1976). A rating of 0–5 has been assigned to each media composition based on olfactory observations of VOCs made by five different individuals. All these tests were performed using a split-plate assay, and after that, to verify whether the pathogens were killed after being fumigated with CEL3 in the most suitable medium, the half-moon agar with CEL3 was removed from the Petri plate, and fungal pathogens remained on the other half-moon side of the agar for the next 5 days. If the pathogen did not grow for the last 5 days, the mycelial plugs from the pathogen were transferred to a fresh PDA medium for observation as to whether the mycelial plugs were recovered within 14 days or not.

### Efficacy of CEL3 on control of fruit rot of cherry by *in vivo* assay

In brief, 50 organic sweet cherries (*Prunus avium*; 8–12 g/cherry) were collected and disinfected by a series of steps: first immersing at 70% ethanol for 1 min and then in 2.5% NaOCl for 3 min, again washing in 70% ethanol for 30 s, finally washing with sterilized distilled water, and finally drying at laminar airflow. Five groups were made, and each group contained 10 cherries and was kept in sterilized transparent plastic boxes (volume 500 ml, length = 12.5 cm, h = 8.5 cm). Group I included only cherry fruits without any infection or endophytic VOC treatment; Group II contained *M*. *fructicola*-infected cherry fruits (as a negative control); in Group III, the treatment group had phytopathogen-infected and endophytic VOC-exposed cherry fruits; Group IV had cherry fruits inoculated with sterile saline water instead of phytopathogen; and Group V had pathogen-infected fruits that were provided with a fungicide treatment instead of VOCs. Systemic imidazole (trade name: Syngenta Amistar Fungicide) at a 2 ml L^−1^ dosage was sprayed over the infected portions. It was considered the positive control. Fruits were inoculated by the wounding method using a sterile needle, and wounds were inoculated with 10 µl of phytopathogen spore solution (10^6^ conidia ml^−1^). In group II (i.e., the treatment group), a lid-less 8-day-old CEL3 culture plate was placed inside the square box, and all the boxes of each group were sealed with Scotch® tape and incubated at 24°C. The development of *M*. *fructicola* was monitored daily, and almost after 6 days, the infection spreads completely and drastically damages the fruit texture. The ability of the endophytic VOCs was evaluated by comparing the disease occurrence in VOC-treated and untreated groups (Pena et al., 2019). Control value (%) = 100 × (radius of the rotted symptom of the *M*. *fructicola* control fruits − radius of the rotted symptom of the treated fruits)/radius of the rotted symptom of the control fruits (Park et al., 2010). The experiments were repeated twice.

### Identification of the endophytic fungal metabolites

#### TLC analysis of the CEL7 metabolites

Partial purification of the bioactive metabolites of the CEL7 extract was performed using thin-layer chromatographic techniques. The fungal extract was prepared as described earlier. The dried fungal extract was resuspended in EA, maintaining a concentration of 20 mg ml^−1^, and 10 µl of EA extract was loaded onto alumina–silica Thin Layer Chromatographic (TLC) plates (MERCK Silica Gel F254) using capillary glass tubes. Acetone (HiMedia) and n-hexane (HiMedia) in a ratio of 2:8 were used as running solvents, and the retention factor was calculated for all the bands under UV light using a Camag UV Cabinet. The bioactive compounds corresponding to each band were scratched and collected, and they were finally dissolved in 1 ml of EA, which was centrifuged (7,000 rpm for 15 min) and evaporated to dryness. The dried components were then dissolved in DMSO at 100 µg ml^−1^ concentration, and the disk diffusion technique measured antifungal action (Wang et al., 2014).

#### GC–MS analysis of the antifungal metabolites

The active fractions obtained from TLC analysis were analyzed using a Gas Chromatography (TRACE 1300)–Mass (ISQ QD Single Quadrupole) Spectrometry (GC–MS) system, Thermo Scientific (USA, Waltham, MA, United States) with an ESI mode. The instrument was configured with a DB-5 Ultra Inert Column (30-m length and 0.25 mm inner diameter) for a 22-min run of a 1 µl sample (a split-less flow) with an injector port and oven temperature of 240 and 50°C, respectively, with a 10°C min^−1^ ramping time up to 260°C with helium as the carrier gas. Tri-plus RSH-based automated injection was done. The flow velocity of the carrier gas was set at 1 ml min^−1^. The ionization source was kept at 250°C with 70 eV of ionization energy and 0.1 kV of ionization current. The mass fragmentation pattern was analyzed by X-Calibur software. The identification of various compounds was based on the SI and RSI values of the best-matched compound in the NIST library (Tomsheck et al., 2010).

Qualitative analyses of the volatiles emitted by the fungal endophyte were done using the solid-phase microextraction (SPME) fiber technique (Mitchell et al., 2009). The endophytic fungus was grown in a 50-ml gas vial (Thermo Scientific, United States), containing 5 ml of modified PDA media (described previously), forming a slant, and was incubated for 8 days at a temperature of 24°C. At first, the inoculated GC-Glass vial was heated at 40°C for 45 min in the incubation cabinet attached to the Tri-Plus RSH autosampler unit of the GC–MS instrument. The incubation cabinet was provided with mild agitation in clockwise and anti-clockwise orientations (five agitations per minute). Then, a baked SPME syringe (Supelco), consisting of 50/30 divinylbenzene/carburen on polydimethylsiloxane on a stable flex fiber, was injected into the GC-Gas vials through the magnet-based rubber cap and exposed to the vapor phase for 45 min. The SPME fiber was then injected into the split-less injection port of the system, which contained the ZB-Wax column (a 30 m diameter, a 0.25 mm inner diameter, and a 0.50 mm thickness). The column was set at 30°C for a 2-min hold, followed by an increase to 225°C at 5°C min^−1^. Helium was used as the carrier gas, with a flow velocity of 1 ml min^−1^. Additionally, the column head pressure was maintained at 50 kPa. The SPME syringe was conditioned at a temperature of 240°C for 25 min under a flow of helium gas (1 ml min^−1^). A 30-second injection time was used to introduce the sample fiber into the system. Bioactive components were identified following the NIST library databases and listed in NIST terminology. The authenticity of the compounds was determined according to the GC/MS of authentic standards (Sigma-Aldrich). Compounds were identified in the NIST library database only when they provided a quality score of 60 or better. The experiments were repeated two times.

### Determination of IC_50_ value of volatile metabolites of CEL3

The artificial mixture of authenticated VOCs was made by mixing the standards of volatiles purchased from Sigma in a particular ratio (following the relative ratio of VOCs emitted from endophytic CEL3). The artificial mixture of VOCs was poured into a pre-sterilized microcup (4–6 mm) and placed in the center of a PDA Petri plate. Agar blocks (5 mm × 5 mm × 5 mm) with pathogenic fungal hyphae were placed on the Petri plate at a 2.5-cm distance from the VOCs containing a microcup and wrapped with two layers of Parafilm. Mycelial growths were measured (in terms of radial growth/colony diameter in centimeters) after 48 h of incubation and continued up to 168 h. Control plates are devoid of synthetic mixes. Three duplicates of the tests were conducted using 2–50 µl of the artificial mixture per 50 mL of air space above the mycelial culture in the PDA plate, and IC50 values were computed.

### Effects of VOCs of CEL3 on the leakage of intracellular components

Leakage of necessary macro-molecules, i.e., the protein, was evaluated following the method of da Rocha (da Rocha Neto et al., 2015) with some modifications. Approximately 100 ml of sterile PDB medium were inoculated with a 5-mm-diameter mycelial plug of the post-harvest pathogens *M*. *fructicola* and *P*. *ultimum*, respectively, in different Erlenmeyer flasks and incubated at 27 ± 2°C with 160 rpm for 48 h in a BOD shaker incubator. Mycelial mass was filtered with Whatman filter paper and washed with sterile water. 1 g of the washed mycelia was resuspended in 100 ml Erlenmeyer flasks containing 30 ml of 0.85% saline. Then, different concentrations of the Artificial-VOC (A-VOC) mixture were added to pathogen-inoculated flasks. The inoculated flasks were re-incubated, and leakage of protein was detected at different time intervals. At each time point, 2 ml of the suspension was centrifuged at 8,000 rpm for 10 min at 4°C to obtain mycelium-free supernatant. Protein contents were measured at 595 nm after 5 min of incubation at room temperature according to the Bradford method. Other than protein, the leakage of intracellular components with absorbance at 260 nm was also evaluated following the methods of Paul et al. (2011). In brief, 1 g of filtrated mycelium from the above-mentioned two pathogens was resuspended in 50 ml of sterile water. Then, A-VOC was added at different concentrations and incubated for different time intervals. Cell-free supernatants were obtained, and OD values were measured at 260 nm.

### Statistical analysis

All experiments were performed in triplicate, and the results are presented as means ± standard errors (SE). Data were analyzed with Prism GraphPad software version 9.2.0 (332) (San Diego, CA, United States). Tukey’s multiple comparison tests were performed in each case to evaluate the statistical significance of the obtained data (P <0.05). Data obtained from the replicates of the study were checked for their normality and were found to be in the normal distribution. They were not transformed and were represented in the form of mean values along with standard errors. A normality test indicated that our data was normal and did not need any transformation. As experiments were repeated, they were checked for homogeneity and then processed further.

## Results

### Identification of the fungal endophytes and evaluation of diversity indices

**Figure 1:**
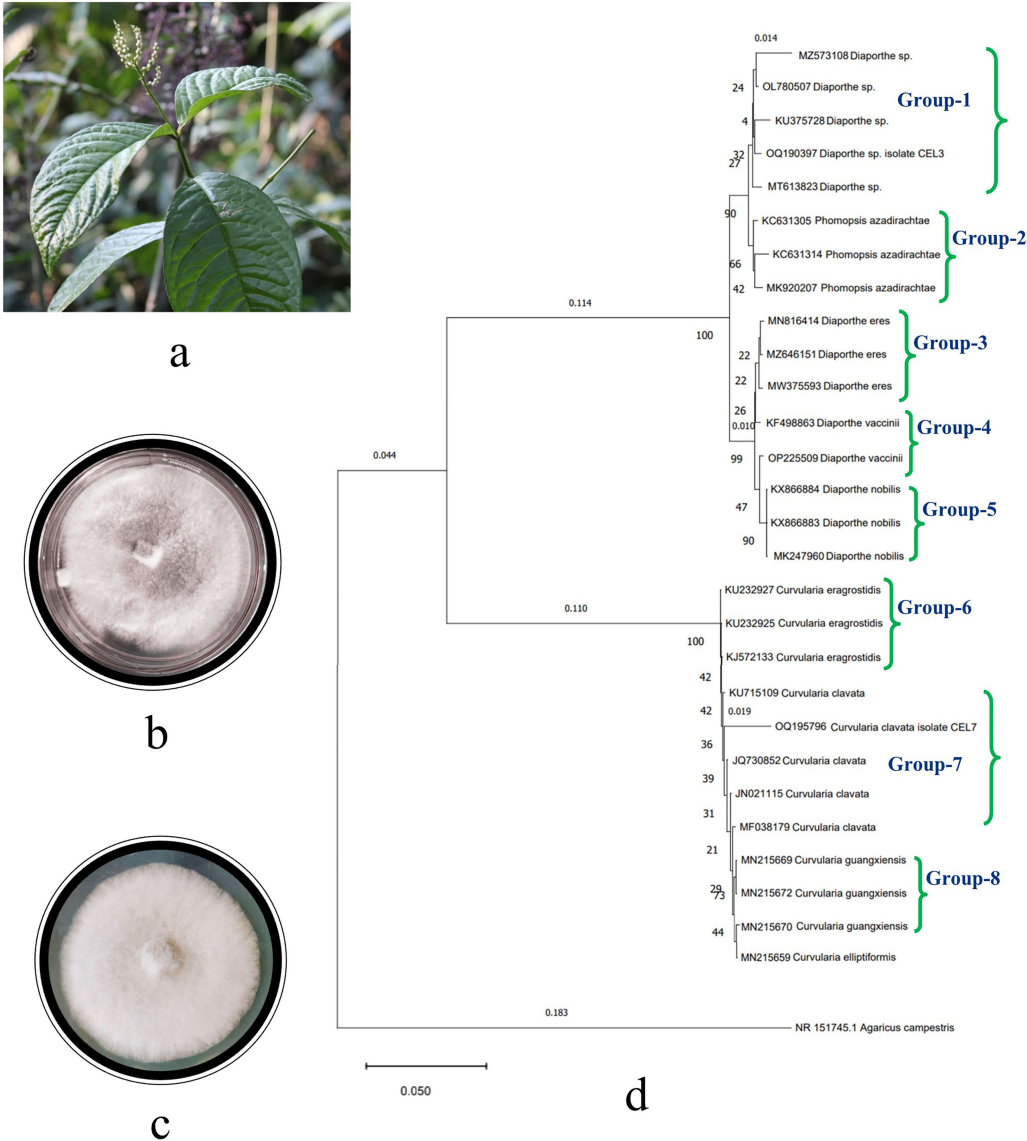
**(A)** Photograph of the host plant, *Chloranthus elatior*, top view of PDA Petri plates of endophytic fungal isolates, **(B)** *Diaporthe* sp. CEL3, **(C)** *Curvularia clavata* CEL7, and **(D)** phylogenetic tree of endophytic fungal isolates CEL3 and CEL7.

A total of 21 fungal endophytes were isolated from the leaf tissues of *C. elatior* (**Figure 1A**). The isolates were non-epiphytic in nature, which was confirmed by the tissue fingerprinting method. Briefly, the aliquots with the wash liquid of the leaves were plated on a PDA, and there was no presence of any fungal taxa. The endophytic fungi were also non-pathogenic, as they were obtained from healthy, disease-free plant leaves. In total, 237 endophytic fungi were isolated from the hundred explants of leaf tissues. Fifteen isolates were identified as *Penicillium* sp., *Aspergillus* sp.1, *Aspergillus* sp.2, *Phoma* sp., *Phomopsis* sp., *Pestalotiopsis* sp., *Alternaria* sp., *Trichoderma* sp., *Curvularia* sp., *Colletotrichum* sp., *Chrysosporium* sp., *Nigrospora* sp., *Helicosporium* sp., *Exerohilum* sp., and *Fusarium* sp. Six isolates remain sterile: CEL1, CEL2, CEL3, CEL5, CEL7, and CEL9. These six sterile isolates remained sterile (**Supplementary Figures 1A, B**) even in leaf carnation agar medium and were unidentified except for the two most efficient antifungal-producing ones, *Diaporthe* sp. CEL3 and *Curvularia clavata* CEL7, with GenBank Acc. Nos. OQ190397 and OQ195796, respectively (**Figures 1B, C**). Endophytic *Pestalotiopsis* sp. and *Helicosporium* sp. were the most frequent colonizers, with a colonization frequency of 18.14% and 15.61%, respectively. Two species of *Aspergillus* (1.26% and 0.84%, respectively) and one species of *Penicillium* (0.42%) were the least frequent. Calculation of diversity indices proves that there is a suitable range of endophytic fungal diversity in the leaf tissues of *C*. *elatior* (**Table 1**). In the cases of CEL3 and CEL7, no distinctive reproductive structure was discovered, and neither isolate’s PDA culture plate contained any diffusible colors. Mycelia were septate and branched for both endophytes as they were rapidly expanding. After four to five days of incubation, they covered 50% of the PDA plate but were slow growing. While CEL3 exhibited a narrow, rough aggregation of pale white mycelium, CEL7 had a dense, milky white mycelial mat. Compared to CEL3, which indicated a slightly irregular colony shape, CEL7 had a smooth, buttery texture.

**Table 1:** Diversity indices of endophytic fungi colonizing leaf tissues of *Chloranthus elatior*.

Diversity indices	Respective values
Simpson’s dominance	0.0971
Simpson’s diversity	0.9029
Species richness	1.37
Shannon-Wiener	2.882
Evenness (E)	0.946

The molecular identification of sterile isolates was done based on two primers, ITS1 and ITS2. The amplified gene products of endophytic isolates were sequenced. A total of 576 and 472 nucleotides were presented in the final datasets of CEL7 and CEL3, respectively. Gaps and missing data were eliminated from the dataset. Sequenced data was compared with the already-available database of GenBank using the BLAST tool, and evolutionary distances were calculated by the neighbor-joining method. Bootstrap analysis was performed to construct the phylogenetic tree, which depicts that the endophytic fungal isolates are phylogenetically related to *C. clavata* and *Diaporthe* sp., respectively (**Figure 1D**), supported by high bootstrap values of 90% and 99% (Saitou and Nei, 1987; Tamura et al., 2004). Cladograms were constructed using 500 bootstrapping repetitions. The phylogram is prepared using 29 isolates grouped into eight sections (Felsenstein, 1985) based on different species of *Curvularia* and *Diaporthe* utilized to set up the tree. Various strains of nine different species of the two isolates were represented in the phylogenetic tree: *Curvularia guangxiensis*, *C*. *elliptiformis*, *C*. *eragrostidis*, *C*. *clavata*. *Diaporthe* sp., *D*. *eres*, *D*. *nobilis*, *D*. *vaccinii*, and *Phomopsis azadirachtae*. Another member of Basidiomycota, *Agaricus campestris*, was taken as the outgroup. The tree starts with MZ5753106 *Diaporthe* sp., along with the other three taxa OL780507, KU375728, and MT613823, and with endophytic isolate OQ190397. These five species constitute Group 1. Groups 2–5 consist of different taxa of *P*. *azadirachtae*, *D*. *eres*, *D*. *vaccinii*, and *D*. *nobilis*, respectively. In Groups 6–8, different species of *Curvularia* are clustered together, respectively. Three strains of *C*. *eragrostidis* and *C*. *guangxiensis* constitute Groups 6 and 8, respectively. Four strains (KU715109, JQ730852, JN021115, and MF038179) of *C*. *clavata* are placed together in Group 7 along with our endophytic isolate OQ195796. The total branch length of the phylogenetic tree was calculated as 0.1543184744. The bioactive endophytic fungal isolates CEL3 and CEL7 evaluated in this study completely match (a 99% query cover) with *C*. *clavata* KU715109 and *Diaporthe* sp., MT613823, and, hence, can be concluded as *C*. *clavata* CEL3 and *Diaporthe* sp. CEL7.

### Broad-spectrum antifungal activity of the endophytic fungal metabolites

#### Optimum emission of CEL3 volatiles and evaluation of the bio-fumigation ability

Out of 21 isolates, one endophytic fungal isolate, CEL3, emits a characteristic scent (a fruity, sweet camphor odor) and inhibits the growth of 10 fungal pathogens from wide taxonomic groups like ascomycetes, basidiomycetes, and oomycetes. The VOCs jad a specific fruity camphor scent due to the abundance of compounds. An increase in relative percentages of verbenol and geraniol emits a strong frequency of sweet camphor odor, which is tested by 10 different healthy lab members with a different olfactory score of 0–10. Lastly, the most appropriate media composition with a high olfactory score was designated as the most suitable VOC-emitting solid media for this isolate, CEL3. The degree of scent emission has been found to be directly proportional to the antifungal activity of the isolate. CEL3 was evaluated for maximum VOC emission on different types of media and a specialized type of media-modified PDA with instantly smashed potato, 40 g L^−1^ dextrose, 20 g L^−1^ agar, and 20 g L^−1^ wheat husk, with an additional 2 g L^−1^ yeast extract, and additional salts were reported to be the most suitable medium for optimum volatile emission (**Table 2**). There was a 7% enhancement in antifungal action as well as a two-fold increase in scent emission under optimized conditions.

**Table 2:** Impact of medium compositions on VOCs production by endophytic fungi CEL3 with qualitative olfactory scores obtained from independent ratings of 0–10, 10 being the maximum.

Medium composition	Independent olfactory Score
PDA	3.33 ± 0.58^a^
Modified PDA with instantly smashed potato (M-PDA)	5 ± 0^b^
OMA	3.67 ± 0.58^a^
PCA	2.33 ± 0.58^a,d^
MEPA	2.67 ± 0.58^a,d^
YEC+ M-PDA (YM-PDA)	7 ± 0^e^
Peptone+ M-PDA	5.67 ± 0.58^b^
Tryptone+ M-PDA	6.67 ± 0.58^b,f^
NH_4_NO_3_+ M-PDA	6.33 ± 0.58^f^
Urea+ M-PDA	6.33 ± 0.58^f^
Bagasse + YM-PDA	7.33 ± 0.58^g,f^
Rice bran + YM-PDA	7.67 ± 0.58^g^
Rice hulls + YM-PDA	8.67 ± 0.58^h^
Soybean meals + YM-PDA	8.67 ± 0.58^h^
Sesame meals + YM-PDA	6.33 ± 0.58^f^
Tea seed pomace + YM-PDA	8.33 ± 0.58^h^
Wheat grains + YM-PDA	8.67 ± 0.58^h^
Wheat husks + YM-PDA	10.33 ± 0.58^i^

The values on the tables are the means ± Standard error (SE) of three replicates. A one-way ANOVA (Tukey’s Multiple Comparison tests) was performed to check the potential statistical differences (P <0.05) between olfactory scores of VOC emission from the endophytic fungi in different media compositions. Similar letters indicate no statistical difference between the data set and different letters (a–i) indicate a valid statistical difference between the data sets.

Out of 10 test fungi, *M*. *fructicola* (78.5%), *P*. *ultimum* (61.9%), *B*. *cinerea* (55.8%), and *R*. *solani* (51.5%) were found to be maximally inhibited by the VOCs (**Supplementary Figures 2A.a–j, 2B.a1–h1**). Other pathogens like *C*. *beticola* (42.6%), *G*. *candidum* (40.4%), and *C*. *ulmi* (35.5%) were also moderately inhibited after 120 h of incubation (**Table 3**). VOCs were ineffective against *A*. *alternata* and *A*. *fumigatus* and minimally inhibited *F*. *oxysporum* (12.2%). CEL3-emitted VOCs were highly biologically selective. The pathogens were transferred to PDA medium post-fumigation experiment, and six phytopathogens, i.e., *M*. *fructicola*, *P*. *ultimum*, *B*. *cinerea*, *R*. *solani*, *C*. *beticola*, and *C*. *ulmi*, showed no growth, which indicates the cidal action of emitted volatiles against these pathogens. The other two VOC-treated fungal pathogens were revived after 48–72 h of incubation, but the growth rate was 20%–45% slower than the control one.

**Table 3:** Antifungal activity of an 8-day-old endophytic CEL3 against selected fungal pathogens.

Fungal pathogens	Inhibition (%) after 120 h of treatment	IC_50_ of artificial atmosphere after 120 h of treatment (µl 50 ml^−1^)	IC_50_ as µl ml^−1^ of air space needed for 50% inhibition
*Rhizoctonia solani*	51.5 ± 2^a^	18.9 ± 1^a1^	0.378
*Alternaria alternata*	No inhibition	65.7 ± 2.4^b1^	1.314
*Fusarium oxysporum*	12.2 ± 1.5^b^	34.8 ± 1.2^c1^	0.696
*Geotrichum candidum*	40.4 ± 2.8^c^	27.6 ± 0.8^d1^	0.552
*Botrytis cinerea*	55.8 ± 1.6^a^	17.0 ± 0.3^a1^	0.34
*Cercospora beticola*	42.6 ± 2.4^c^	24.5 ± 0.4^d1^	0.49
*Aspergillus fumigatus*	No inhibition	No inhibition	–
*Ceratocystis ulmi*	35.5 ± 1.6^d^	31.6 ± 1.8^c1^	0.632
*Pythium ultimum*	61.9 ± 2.8^e^	14.7 ± 1.5^a1^	0.294
*Monilinia fructicola*	78.5 ± 4.2^f^	09.8 ± 0.8^e1^	0.18

The values on the tables are the means ± standard error (SE) of three replicates. A one-way ANOVA (Tukey’s Multiple Comparison tests) was performed to check the potential statistical differences (P <0.05) between the antifungal activity of the VOCs of endophytes against different pathogens (a–f). Also, the IC_50_ values of the artificial atmosphere were checked through the same technique (a1–e1). Similar letters indicate no statistical difference between the data set, and a different letter indicates a valid statistical difference between the data sets.

#### The antifungal activity of CEL7 cell-free metabolites

Cell-free culture extracts of isolate CEL7 exhibit maximum antifungal activity against fungal pathogens in the agar well diffusion technique. Pathogenic fungi *M*. *fructicola*, *R*. *solani*, *B*. *cinerea*, and *C*. *beticola* were maximally inhibited, whereas the endophytic metabolites moderately inhibited the growth of other pathogens (**Supplementary Figures 3A–H**). The MIC of CEL7 metabolites against the ten fungal pathogens ranged from 100 µg ml^−1^ to 2 mg ml^−1^ (**Table 4**).

**Table 4:** Minimum inhibitory concentrations (MIC) of endophytic fungal metabolites of CEL7 against fungal pathogens.

Sl. No.	Name of the fungal pathogen	MIC value (µg ml^−1^)
1	*M*. *fructicola*	233.33 ± 19.07^a^
2	*R*. *solani*	400 ± 0^b^
3	*B*. *cinerea*	533.33 ± 58.11^c^
4	*C*. *beticola*	633.33 ± 19.07^d^
5	*A*. *alternata*	1,500 ± 0^e^
6	*F*. *oxysporum*	833.33 ± 58.11^f^
7	*G*. *candidum*	1,200 ± 0^g^
8	*A*. *fumigatus*	2,000 ± 0^h^
9	*C. ulmi*	1,100 ± 0^g^
10	*P. ultimum*	700 ± 0^d^

The values on the tables are the means ± standard error (SE) of three replicates. A one-way ANOVA (Tukey’s Multiple Comparison tests) was performed to check for potential statistical differences (P <0.05) between the antifungal activity of the endophytic extract against different pathogens. Similar letters indicate no statistical difference between the data sets, and a different letter indicates a valid statistical difference between the data sets.

### Fumigation activity of CEL3 against cherry fruits

**Figure 2:**
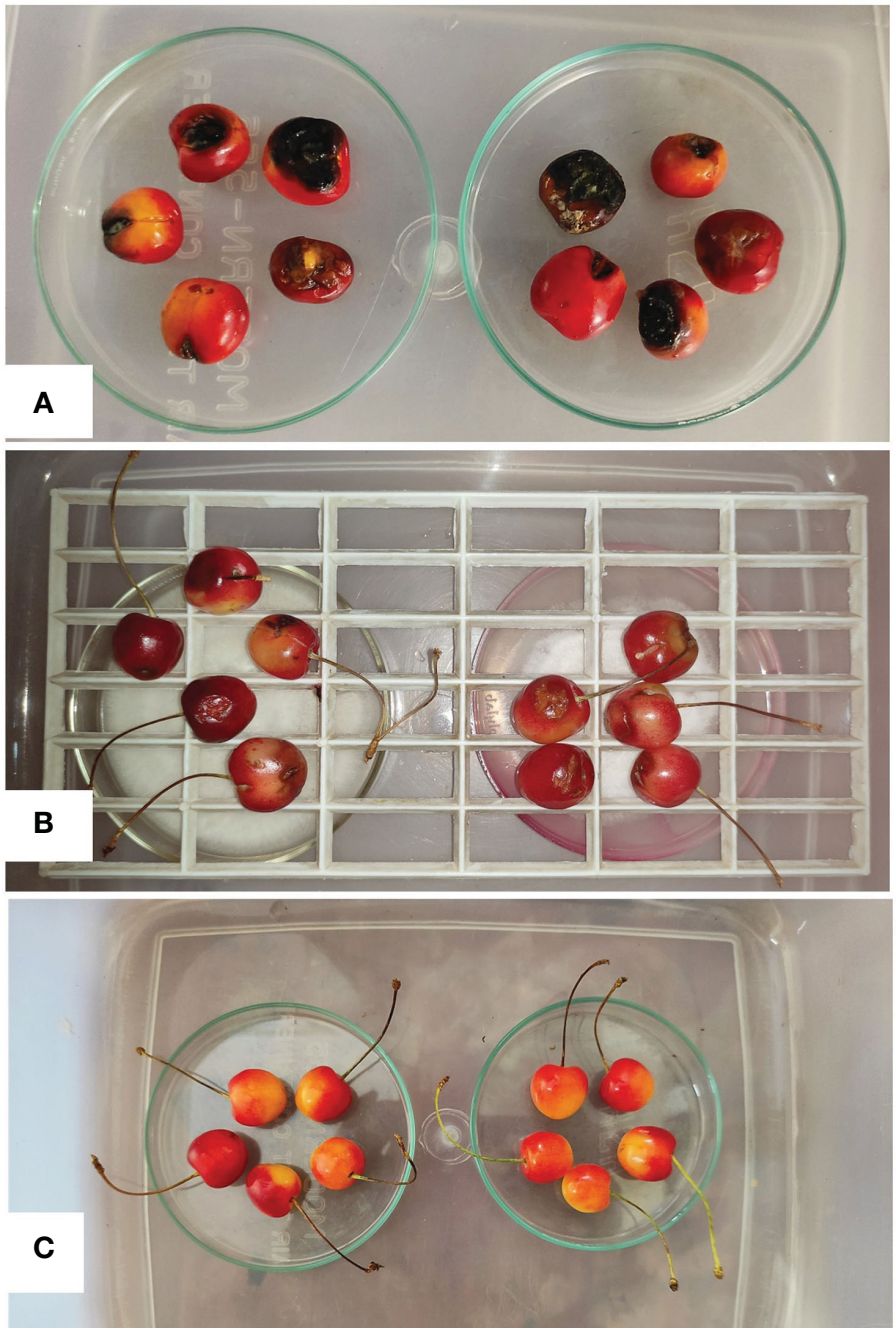
**(A)** Disease symptoms on cherry fruits inoculated with *M*. *fructicola* pathogen only (non-VOC treated set), **(B)** Cherry fruits infected with *M*. *fructicola* pathogen and exposed to *Diaporthe* sp. CEL3 VOCs for 8 days appear to be healthier, and less disease infected in comparison to the non-VOC-treated set, and **(C)** Cherry fruits in control condition without any inoculation of pathogen and application of CEL3 volatiles.

*M*. *fructicola*-infected (Group II-control condition, without any endophytic VOC treatment) cherry fruits were found to be extremely damaged due to the pathogenic invasion; fruits started to rot quickly, and pathogens occupied the internal tissues of the tested fruits. Black-colored rotten patches appeared all over the fruit, especially at the sites of pathogen inoculation. The fruits started to decay after the 4th day of inoculation with the *M*. *fructicola* pathogen. All 10 fruits were damaged by the fungal attack, and the natural cherry red color turned pale yellow first, and then black spots occupied the fruits (**Figure 2A**; **Supplementary Figure 4A**). Whereas the fruits that were first inoculated with the fungal pathogen and then exposed to endophytic fungal (CEL3) VOCs (i.e., Group III) were found to be in healthy situations compared to the non-VOC-treated ones (**Figure 2B**). **Figure 2C** shows the cherry fruit control group that received no treatment. **Supplementary Figure 4B** shows cherry fruits that have been infected with the *M*. *fructicola* pathogen and treated with a systemic fungicide. A mild degree of pathogenic invasion was also located here, but the tissues did not degrade, and no rotten black patches were visible even after 8 days of inoculation. Only the tissue damage was located at the site of pathogen infection, but that too was a very preliminary invasion with minute damage. CEL3 fumigated fruits were microscopically observed; no necrotic tissues were found, and no such topical tissue degradation symptoms were reported. So, the endophyte CEL3 possesses unique bioactive antifungal VOCs with fumigating properties and can be commercially used for post-harvest disease management of economically valuable cherry fruit. The viability of the pathogen after VOC treatment was evaluated by attempting re-isolation of the inoculated pathogen from the cherry fruits, and it has been found that *M*. *fructicola* was isolated from non-VOC-treated cherry fruits, but there was a 10% isolation frequency of the inoculated pathogen from the VOC-treated cherry fruits, i.e., out of 10 tested fruits, the viable pathogen was isolated from only one cherry fruit. The results clearly indicate that there was 90% inhibition of pathogenic growth *in vivo* after VOC treatment.

### Profiling of antifungal metabolites

The ethyl acetate fraction of CEL7 cell-free metabolites appeared to have four different bands under UV light (254 nm and 366 nm) after TLC analysis. All four fractions were tested for antifungal action, and band C with an Rf value of 0.76 held maximum antifungal action (**Supplementary Figure 5A**). Antifungal metabolites present in band C were identified by the GC–MS-NIST library (**Supplementary Figure 5B**). The major compounds detected are actinobolin, bactobolin, Pyrazole 4,5 imidazole, 1-formyl 3 ethyl, Phenol, 4-[2-(methylamino) ethyl], and -(2-aminoethyl)-1H-imidazole-2-carbaldehyde (**Table 5**).

**Table 5:** Compounds identified from band-C of EA fraction of *Curvularia clavata* CEL7 by GC-MS-NIST library.

Sl. No.	Emitted volatiles	RT (min)	Area (%)	MW
1	(2-aminoethyl)-1H-imidazole-2-carbaldehyde (C_6_H_9_N_3_O)	2.29	33.11	139
2	Phenol, 4-[2-(methylamino) ethyl] - (C_9_H_13_N_O_)	5.54	6.29	151
3	Actinobolin (C_13_H_20_N_2_O_6_)	6.53	3.97	300
4	Chloroacetic acid, 2-ethylhexyl ester (C_10_H_19_ClO_2_)	10.66	6.29	206
5	Bactobolin (C_14_H_20_Cl_2_N_2_O_6_)	12.46	11.92	382
6	Pyrazole 4, 5 imidazole, 1-formyl 3 ethyl (C_12_H_16_N_4_O_5_)	13.97	17.21	296
7	O-Methylisourea (C_2_H_6_N_2_O)	17.31	3.97	74
8	6-Nitro-3-chlorophenol (C_6_H_4_ClNO_3_)	22.47	9.93	173
9	Phenol, 2,4,6-tri-tert-butyl- (C_18_H_30_O)	24.70	7.29	262

In total, 11 emitted volatile metabolites were detected from an eight-day-old endophytic fungus CEL3, and the major components were reported to be trans-verbenol (C_10_H_16_O): 32.25%, geraniol (C_7_H_6_O_2_): 30.32%, and trans-ocimenol (C_10_H_18_O): 12.90% at a retention time of 19.95 min, 18.81 min, and 15.69 min, respectively (**Table 6**). Other volatiles were mentha-4,8-diene (C_10_H_16_), 1,2-butadiene, 3-phenyl (C_10_H_10_), and the methyl ester of citronellic acid (C_11_H_20_O_2_) (**Supplementary Figure 6**). The chemical structures of different antifungal metabolites synthesized by CEL3 and CEL7 are recorded in **Supplementary Figure 7**. The major component, trans-verbenol emits a minty, sweet camphor-like smell, and the second major compound, geraniol, holds a rose-like citrus and fruity odor. So, the overall emission represents a fruity, sweet camphor odor.

**Table 6:** GC/MS-SPME analysis of the volatile organic compounds (VOCs) emitted by an 8-day-old endophytic *Diaporthe* sp. CEL3 grown on a modified PDA medium.

Sl. No.	Emitted volatiles	RT (min)	Area %	MW
1	Citronellic acid, methyl ester (C_11_H_20_O_2_)	13.47	3.87%	184
2	Mentha-4,8-diene (C_10_H_16_)	13.66	5.16%	136
3	Trans-ocimenol (C_10_H_18_O)	15.69	12.90%	154
4	cis-Isopiperitenol (C_10_H_16_O)	15.90	3.87%	152
5	2-Octenal (C_8_H_14_O)	17.54	1.93%	126
6	Geraniol (C_7_H_6_O_2_)	18.81	30.32%	122
7	1,2-Butadiene, 3-phenyl (C_10_H_10_)	18.97	5.48%	130
8	Trans-verbenol (C_10_H_16_O)	19.95	32.25%	152
9	9-Cedranone (C_15_H_24_O)	22.10	1.29%	220
10	1-nonanol (C_9_H_20_O)	22.48	2.25%	144
11	1-Naphthylmethanol (C_11_H_10_O)	22.83	0.64%	158

### Determination of IC_50_ value of the artificial atmosphere

**Figure 3:**
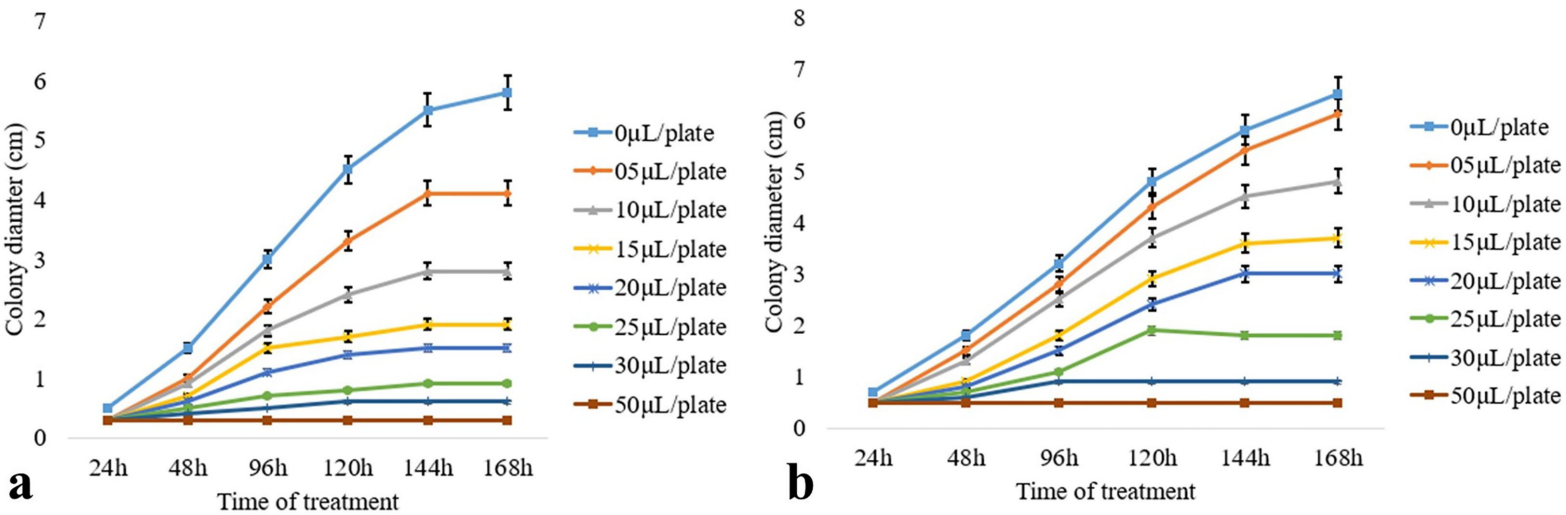
Effect of artificial volatiles on the mycelia growth, i.e., colony diameter of two post-harvest fungal pathogens **(A)** *M*. *fructicola* and **(B)** *P*. *ultimum* at different time intervals. The values on the graphs are the means ± standard error (SE) of the three replicates. Tukey’s multiple comparison test was performed, and there was a significant statistical difference between the control and treated sets (P <0.05).

Antifungal volatile organic compounds produced by the endophytic fungi *Diaporthe* sp. CEL3 (discussed previously) were detected using GC–MS. The standards of the major components (like verbenol, geraniol, and ocimene) were bought from Sigma Aldrich, and they were mixed in a proper ratio (as emitted by the endophytic fungi). These artificial mixtures of volatiles (A-VOCs—artificial VOCs) were evaluated for their antifungal activity against the same set of pathogens. The IC_50_ (in the complete air space of the test Petri plate) value of that artificial mixture was calculated, which ranged between 9.8 µl 50 ml^−1^ and 65.7 µl 50 ml^−1^ for 50% inhibition of fungal pathogens (**Table 3**). At an A-VOC exposure of 135 µl 50 ml^−1^ for 144–168 h, all the fungal pathogens were inhibited completely, except *A*. *fumigatus*. The degree of action of the artificial atmosphere on inhibiting fungal growth varies from one organism to another. Thus, volatiles are highly selective. *M*. *fructicola*, *P*. *ultimum*, *B*. *cinerea*, and *R*. *solani* were 50% inhibited even at a little dose of VOCs (9.8, 4.7, 17, 18.9 µl 50 ml^−1^, respectively), but for 50% growth inhibition of *C*. *ulmi*, *F*. *oxysporum*, and *A*. *alternata*, 31.6, 34.8, and 65.7 µl 50 ml^−1^ of VOCs were needed, respectively. It proves that some pathogens are highly sensitive to VOCs, whereas others are less sensitive. *A*. *fumigatus* was resistant to both the artificial mixture (A-VOCs) and the fungus-emitted VOCs. *A*. *alternata* growth was not at all hampered by fungus-emitted volatiles whereas, artificial VOCs at a high value of 65.7 µl 50 ml^−1^ stopped its growth. Growth kinetics (in terms of inhibition of the colony diameter, i.e., radial growth) of the two tested pathogens that were maximally inhibited (*M*. *fructicola* and *P*. *ultimum*) by the minimum exposure to the artificial mixture of VOCs are illustrated in **Figures 3A, B**. There was maximum inhibition of fungal growth at 50 µl of A-VOC exposure. But as we discussed earlier in this section half of the growth ceased at their respective IC_50_ dosages (**Figure 3A**—for *M*. *fructicola* IC_50_- 10 µl and **Figure 3B**—for *P*. *ultimum* IC_50_- 15 µl) after a minimum exposure of 120–144 h.

### VOCs of CEL3 cause leakage of intracellular macromolecules from post-harvest fungal pathogens

**Figure 4:**
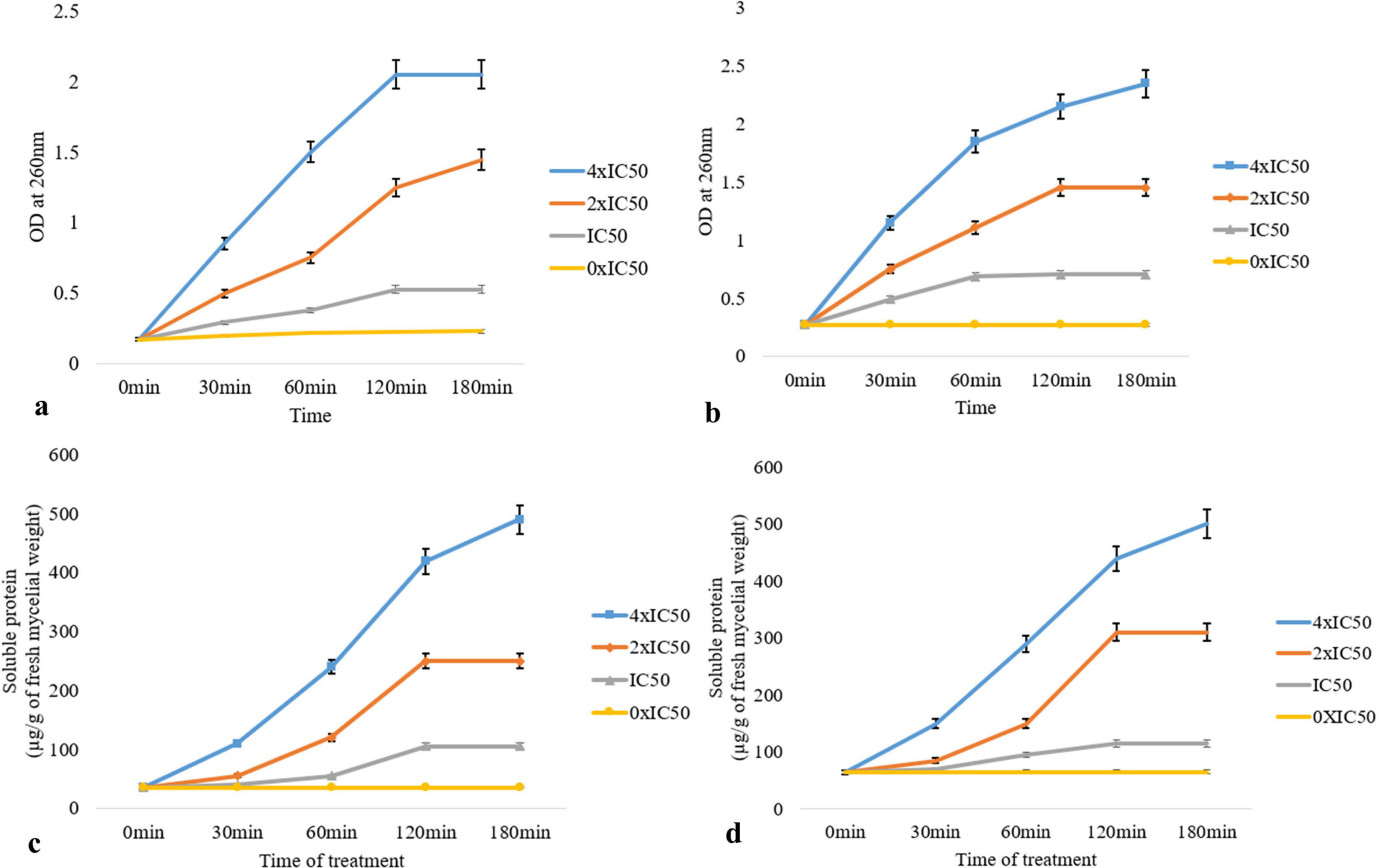
Effect of different concentrations of artificial volatiles on leakage of intracellular components detected at OD_260 nm_ **(A, B)** and soluble proteins **(C, D)** from mycelial cells of *M*. *fructicola* **(A, B)** and *P*. *ultimum* **(C, D)**. The values on the graphs are the means ± standard error (SE) of the three replicates. Tukey’s multiple comparison test was performed, and there was a significant statistical difference between the control and treated sets at different concentrations of volatiles and at different incubation periods (P <0.05).

The leakage of supernatants (at OD_260 nm_) from the A-VOC-treated mycelium of two post-harvest fungal pathogens was found to be gradually increased with treatment time as well as the concentration of A-VOCs in comparison to the control (0xIC_50_). At the 4× IC_50_ dosage, there was maximum leakage of intracellular compounds from both the fungal pathogens, i.e., *M*. *fructicola* and *P*. *ultimum*, respectively (**Figures 4A, B**). The value of optical density at 260 nm in the 120 min test group was significantly higher than that of the 0–60 min test group (P <0.05) in all three treatment concentrations of IC_50_, 2× IC_50_, and 4× IC_50_. So, it could be inferred that with the passage of treatment time, the release of cell components increased, with the highest rate attained at 120 min. **Figures 4C, D** illustrate the protein leakage profile of the A-VOC-treated fungal mycelium of two post-harvest pathogens. Similar to the leakage of intracellular components at OD_260_, the leakage of soluble proteins into the extracellular environment increased parallel to the incubation time. A treatment with IC_50_, 2× IC_50_, and 4× IC_50_ doses led to leakage of 115, 310, and 439 soluble proteins (mg/g fresh mycelium) from *M*. *fructicola* fungal cells. Whereas in the control set, only 35 mg of protein leakage was detected after 120 min of incubation. A similar type of protein leakage profile is reported in the case of another test pathogen, *P*. *ultimum*.

## Discussion

Endophytic microbes, especially endophytic fungi, are potent producers of plant-protective bioactive compounds that hold immense utility in the field of agriculture, specifically in controlling fungal-pathogen-related post-harvest diseases of crop plants by adopting direct and indirect routes (Rai et al., 2014). There are two major types of phytoprotective metabolites that are synthesized by endophytes: volatile organic compounds and non-volatile soluble/insoluble short- and long-chain metabolites. Here, both the volatile and non-volatile endophytic fungal metabolites have been evaluated for their antifungal activity, with a special emphasis on the mycofumigation ability of the endophytic volatiles. VOCs are organic low molecular mass (100–500 Daltons) carbon-containing small compounds (up to C20) that have a high vapor pressure at room temperature and are generally emitted from various kingdoms of living organisms, plants, animals, bacteria, and fungi in little or significant amounts and serve several unique bioactivities in the field of agriculture and pharmaceuticals such as antimicrobial, anti-proliferative, anti-larval, plant growth promotion, antifungal, etc. (Kesselmeier and Staudt, 1999; Korpi et al., 2009; Schulz-Bohm et al., 2017; Santra and Banerjee, 2020). As fungal diseases are the most lethal infectious agents of the agroecosystem that disrupt the overall crop productivity and adversely affect the agrarian economy, the focus of this article is on the antifungal principles of volatiles. An estimated 10%–15% of the world’s major crops are lost to plant diseases each year, resulting in direct economic losses of up to hundreds of billions of dollars. Approximately 70%–80% of these harms are brought on by pathogenic fungi (Chatterjee et al., 2016; Li et al., 2017, Peng et al., 2021). Fungal diseases are major hindrances to the smooth running of sustainable agricultural practices (Marin-Menguiano et al., 2019). Traditional methods of controlling fungal pathogens are getting more resistant day by day, and there is an urgent urge for the formulation of novel strategies with deep ecological approaches. Natural products are the best solution to address this issue, but other than plant-based products or phyto-metabolites, the fungal endophytes develop eco-friendly and economically feasible agricultural products (Lugtenberg et al., 2016). Like endophytic fungi, *Muscodor albus* and its different volatile antifungal-producing strains have been previously reported as potent agents of myco-fumigation and have well-established (US patent 6,911,338) use as a bio-control agent for insect pests, nematodes, arthropods, post-harvest fungal pathogens, and human pathogenic bacteria (Strobel et al., 2001, Saxena and Strobel, 2021).

According to the current study, cherry fruits infected with *M*. *fructicola* are protected from rotting by the sterile fungal endophytes of the ethnomedicinal plant *C*. *elatior*, which also produces volatile and non-volatile antifungal compounds. The Passighat woodlands in Arunachal Pradesh, Northeastern India, a region of tropical rain forest, were where *C*. *elatior* was discovered. Tropical rain forests have been extensively investigated for the scavenging of bioactive novel endophytes throughout the previous twenty years of endophyte biology study, and each time the findings were fascinating. (Meshram et al., 2016; Saxena and Strobel, 2021). The tropics are an ideal choice, particularly when the emphasis is on volatile organic compound-generating isolates. For this reason, the northeastern Indian flora has been chosen as a source of endophytic fungi. Endophytes and their hosts are involved in a successful functional symbiosis and are popularly designated as endophyticism (Hereme et al., 2020).

As a result, the long-term co-evolution of the host and endophyte has focused on interspecies gene sharing using both horizontal and vertical gene transfer roots. Endophytic fungi consequently generate unique bioactive substances that are comparable to or superior to the host metabolome (Khare et al., 2018). Science has debated the real endophyte character of fungal invaders, and in this study, we focused on Class 3 endophytes, which are efficient protectors against a variety of abiotic and biotic stresses (Rodriguez et al., 2009). A total of 332 fungal colonies from 555 explants of *C. japonicus* were reported from an investigation into the diversity of its fungal endophytes. With the use of ITS sequencing, 130 strains were discovered. These strains are members of the phylum Ascomycota, Basidiomycota, and Mucoromycota, which includes five classes: Agaricomycetes, Dothidiomycetes, Eurotiomycetes, Mucoromycetes, and Sordariomycetes. *Colletotrichum*, *Aspergillus*, and *Diaporthe* were the three major genera, with relative abundances of 60.54%, 11.75%, and 9.34%, respectively. The isolates demonstrated potent antifungal activity as well as biological control abilities. With values of 56 and 2.7076 for the Shannon-Wiener (H) and species richness (S) diversity indices, respectively, it may be concluded that the host tissues contain a significant number of fungal endophytes. This study’s report on the diversity of endophytes observed in *C*. *elatior* mostly accords with earlier findings (An et al., 2020).

Indian medicinal plants are always incredibly diverse when it comes to endophytic fungi, especially those that originate from untouched regions with an abundance of greenery. Nalini et al. (2014) reported 1,052 fungal endophytes from seven ethnomedicinal plants collected from the Western Ghats: *Tylophora asthmatica*, *Rubia cordifolia*, *Plumbago zeylanica*, *Phyllanthus amarus*, *Eryngium foetidum*, *Centella asiatica*, and *Zingiber* sp. In comparison to below-ground tissues (19.22%), which contain 31 fungal taxa from ascomycetes (3%), coelomycetes (65%), and hyphomycetes (32%), the above-ground tissues have a higher diversity of endophytes (80.37%). *Acremonium*, *Colletotrichum*, *Chaetomium*, *Fusarium*, *Myrothecium*, *Phomopsis*, and *Pestalotiopsis* were the principal genera mentioned. A total of 72 different bacterial endophytes were found in 24 Western Ghats medicinal plants, according to Webster et al. (2020). There are several bioprospecting potentials for the pharmaceutical sector among the vast flora of fungal endophytes found in the plants of the Thar Desert (Yadav and Meena, 2021). A variety of endophytes found in Asian medicinal plants create a wide range of bioactive compounds that are widely used in agriculture and pharmaceuticals (Sharma et al., 2020; Jagannath et al., 2021; Singh et al., 2022). Here, research has been done on *C*. *elatior*, a perennial Himalayan subshrub that is endemic to China and the northeastern provinces of India and is a member of the Chloranthaceae family. (Sun et al., 2012). The leaf, root, and stem bark contain terpenoid-rich phytochemicals that have hepatoprotective, anti-tumor, antibacterial, anti-inflammatory, and antifungal properties (Wang et al., 2015). The plant was chosen because it has a wide range of uses in conventional folk medicine. There are limited reports on endophytes in this genus, and *C*. *elatior* endophytes have not yet been shown to produce antifungal VOCs (An et al., 2020).

Here, it was discovered that two sterile endophytic isolates, known as *Curvularia* sp. CEL7 and *Diaporthe* sp. CEL3, could produce antifungal metabolites. Species of *Penicillium*, *Aspergillus*, *Phoma*, *Phomopsis*, *Pestalotiopsis*, *Alternaria*, *Trichoderma*, *Colletotrichum*, *Chrysosporium*, *Nigrospora*, *Helicosporium*, *Exerohilum*, and *Fusarium* are among the other isolates that produce spores or conidia. Different species of these fungal taxa are known to infect a variety of agricultural crops, and they have been randomly reported as endophytes as well (Chagas et al., 2013; Thanabalasingam et al., 2015; Suryanarayanan et al., 2018; Chatterjee et al., 2022; Santra and Banerjee, 2022a; Santra and Banerjee, 2022b; Santra et al., 2022). Metabolites of endophytic fungi *Diaporthe* sp., an isolate of *Pinus densiflora*, showed antibacterial, antidiabetic, and anticancer action, according to Saravanakumar et al. (2021). Endophytic *Phoma* sp., and *Phomopsis* sp. isolated from *Larrea tridentata* and *Odontoglossum* sp., respectively, have been reported as potent VOC producers with antifungal activity against fungal pathogens. The major emitted volatile metabolites were 1-Propanol, 2-methyl-, 2-propanone, sabinene, 1-Butanol, 3-methyl, gymnomitrene, beta-acoradiene, dodecanoic acid, hexadecenoic acid, 1-Butanol, 3-methyl-, acetate, propanoic acid-2-methyl, α-Humulene, β-Selinene, aromadendrene, α-amorphene, β-himachalene, and cadinene (Singh et al., 2011; Strobel et al., 2011). These endophytes emitted maximum VOCs in modified culture media with potato dextrose agar prepared from instantly smashed potatoes, along with yeast extract and additional salts. Another endophytic fungus, *Curvularia eragrostidis* HelS1, an isolate of *Helicteres isora*, synthesizes a broad spectrum of volatile and non-volatile metabolites with a wide range of antibacterial and antifungal activity (Santra and Banerjee, 2022c).

Here, the endophytic *Diaporthe* sp. CEL3 isolated from leaf tissues of the explant produces rose-like fruity, sweet-citrus-odor geraniol at sub-major levels in a basal PDA medium, herbal, sweet-scented transocimenol, and minty, sweet verbenol at maximum percentages. To get the highest possible olfactory score of ten, instant mashed potatoes with added dextrose, yeast extract, and wheat husk at doses of 150 g L^−1^, 20 g L^−1^, and 20 g L^−1^, respectively, were employed. In contrast, HelS1 is said to release azulene and naphtha as the main substances in the changed PDA medium. An endophytic *Nodulisporium* sp. inhibited the pathogen, *Penicillium digitatum* Cmep-13, by producing 2-ethyl-2-hexenal and 2,4-8 dimethyl-1,3-cyclopentanedione in a specialized formulation on bagasse (Yeh et al., 2021). Mitra et al. (2023) reported the antifungal activity of fruity-scented volatiles emitted from endophytic *G. candidum* PF005 against stored grain pathogens *R. solani* and *Curvularia oryzae* in a low-cost solid substrate medium. Other than fungi and endophytic bacteria, *Burkholderia pyrrocinia* also inhibits the poplar canker pathogens *Cytospora chrysosperma*, *Phomopsis macrospora*, and *Fusicoccum aesculin* through the emission of volatile metabolites (dimethyl disulfide, benzothiazole, dimethylthiomethane, and phenylacetone) and improves the antioxidant defense system *in vivo* (Liu et al., 2020). Al-Rashdi et al. (2022) documented the anti-phyto-pathogenic activity of fungal endophytes of *Aloe dhufarensis*–*Sarocladium kiliense* (SQUCC-F2-2) and *Penicillium oxalicum* (SQUCC-F3-1) against *Fusarium* sp. and *Cladosporium* sp., and the major antifungal metabolites were tetradecanoic acids, furfuryl alcohol, 1,2-diols, and amide, 2-furanmethanol, and dodecanoic acid. The test fungi in the present study are dreadful pathogens of cash crops, fruits, and vegetables, including wine grapes, rapeseed, tomato, tangerine, tobacco, strawberry, cherry, and wheat. These fungi cause necrosis, damping off, wilt, root rot, fruit rot, leaf spot, etc., which upsets the balance of the agricultural economy (Verma, 1996; Holmes and Clark, 2002; Agrios, 2005; Williamson et al., 2007; Michielse and Rep, 2009; Meena et al., 2017). Six of the infections that were tested—*M*. *fructicola*, *P*. *ultimum*, *B*. *cinerea*, *R*. *solani*, *C*. *beticola*, and *C*. *ulmi*—were killed by the volatiles of CEL3, while two other pathogens—*G*. *candidum* and *F*. *oxysporum*—regained viability but had slowed growth. *A. fumigatus* and *A. alternata* were not successfully treated with the volatiles of CEL3. According to earlier reports, the endophytic fungus *Daldinia* cf. *concentrica*, *M. albus*, *M*. *camphora*, and *M*. *ghoomensis*, as well as their VOCs, effectively removed these two isolates (Ezra and Strobel, 2003; Meshram et al., 2015; Liarzi et al., 2016; Meshram et al., 2017). After 48–144 h of exposure, volatiles from *M*. *albus*, *Phoma* sp., and *Phomopsis* sp. may kill various classes of fungal pathogens, including *Phytophthora cinnamomi*, *P*. *palmivora*, *Verticillium dahliae*, *Sclerotinia sclerotiorum*, *F. solani*, *P*. *ultimum*, *B*. *cinerea*, *R*. *solani*, *C*. *beticola*, and *C*. *ulmi* (Banerjee et al., 2010b; Singh et al., 2011; Strobel et al., 2011; Banerjee et al., 2014). The results of this investigation are essentially consistent with recent research on the biological activity of VOCs released by endophytes. So, endophyte-derived volatiles are of a diverse chemical nature and serve a variety of biological functions. To avoid or minimize financial losses caused by these fungal pathogens, preventative steps are taken. Millions of dollars are also spent to tackle these diseases, which include crop rotation, choosing plants that are resistant to them, and using chemical fungicides (Mirzaazimova et al., 1994; Raza et al., 2016). Though soil-borne fungal infections are frequently controlled with synthetic fungicides, it has been demonstrated that these worsen soil quality. Also increase soil-borne plant diseases, phytotoxicity, food contamination, and environmental pollution (Aktar et al., 2009). So, on the one hand, toxic-harmful chemical formulations are widely applied, which are getting incorporated into the food chain and causing biomagnification, but these chemicals cannot even prevent 7% to 24% losses in the yield of revenue crops (Bailey and Lazarovits, 2003). In the present global context, organic solutions are always chosen to increase reliance on sustainable agricultural practices (Zubrod et al., 2019). Consequently, there is a high demand for microbial biocontrol agents (MBA), and among the various mechanistic strategies used by these agents to control plant pathogens, including priming or enhancing plant tissues, modifying pathogen growth conditions, hyper-parasitism, antibiosis, and enzymatic hydrolysis of pathogen structures, antifungal action through emitted volatiles is the best choice (Spadaro and Droby, 2016; Ghorbanpour et al., 2018). Therefore, the fungal isolate CEL3 can be employed as a beneficial biocontrol agent with strong myco-fumigation potential instead of xenobiotic antifungals that have significant adverse effects.

As released by the endophyte CEL3, we also assessed the fungi-toxic effects of the synthetic combination composed of standard volatiles generated at certain ratios. After 120 h of exposure, it is discovered that the imitated mixture is antifungal in nature, with an IC_50_ value of 9.8–65.7 L 50 ml^−1^. The same group of pathogens is known to be blocked by the synthetic environment that resembles the endophytic *Phomopsis* sp. VOCs after 48 h of exposure (Singh et al., 2011). Some of the volatiles generated by some endophytes have been reported earlier from other endophytes and in the composition of phytochemicals or essential oils, but others are novel and not yet available in the form of standards (Sharifi-Rad et al., 2017; Andrade et al., 2021). Considering this, endophytes do produce unique compounds that have a variety of uses in the sectors of agriculture and medicine. (Baiome et al., 2022).

Eight fungal diseases were successfully combatted by fungi volatiles, with *M. fructicola* showing the greatest degree of inhibition. In cherry fruits infected with *M*. *fructicola*, volatiles of CEL3 also reduced the occurrence of fruit rot. Different species of this pathogen, i.e., *M*. *laxa*, *M*. *fructigena*, and *M*. *fructicola*, are the causal agents of the rot of valuable stone fruits: peach (*Prunus persica*), nectarine (*Prunus persica* var. *nucipersica*), almond (*Prunus dulcis*), apricot (*P*. *armeniaca*), plum (*P*. *domestica*), apple (*Malus pumila*), pear (*Pyrus communis*), sour cherry (*P*. *cerasus*), and sweet cherry (*P. avium*) (Batra, 1991). Approximately 10% of the world’s stone fruit market (17MT, EPPO, 2012), or 1.7 M/year, is lost due to monilia infection, and even copper-based fungicides lose their effectiveness. On commercial organic farms, brown rot specifically accounts for approximately 75% of crop loss (Larena et al., 2005; Xu et al., 2007). So, there is an urgent requirement for sustainable biocontrol agents, and fungal volatiles have played a major role here. Only 10% of fungal pathogens from fruits treated with VOCs of CEL3 survived; as a result, VOCs were deadly and killed 90% of the fungal pathogens. *In vivo* application of endophytic fungi and utilization of the emitted volatiles in mycofumigation is a popular technique for managing post-harvest infections. *Muscodor brasiliensis* sp. nov. was isolated from the Brazilian medicinal plant *Schinus terebinthifolius* and was reported to restrict the growth of *P. digitatum* (the causal agent of the green mold of citrus) in orange fruits through its emitted volatile (Pena et al., 2019). Volatiles of the endophytic fungus *Nodulisporium* sp., an isolate of *Peperomia dindygulensis* leaf, in a special type of bagasse media (PDL005) controlled the *P*. *digitatum* infection in orange (Yeh et al., 2021).

Microbial volatiles are known to be both biocidal and biostatic (Ye et al., 2020). The current finding indicates that CEL3 volatiles have fungicidal effects on *M*. *fructicola*. To limit *M*. *fructicola* infection in cherry fruits, many targeted pathogen-specific mechanistic techniques may interact with the VOCs. According to reports, VOCs disintegrate the outermost anatomical barriers, such as the cell wall and cell membrane, and progressively assemble and adhere to the hyphal cell wall of harmful fungi (Ye et al., 2020). They soon start to disintegrate, their permeability gets increased, and as a result, intracellular macromolecules and housekeeping enzymes, i.e., nucleic acid and proteins, get leaked, leading to the shutdown of the pathogens’ biochemical machinery (Oonmetta-aree et al., 2006). Other than that, a significant amount of reactive oxygen species (ROS) gets accumulated inside the volatile treated cells, and high ROS levels hamper oxidative phosphorylation and affect the permeability of mitochondrial membranes (Robson, 2006; Huang et al., 2019). In some instances, pathogens treated with VOCs have their melanin or other important pigment manufacturing pathways disrupted, which causes radiative and photo-oxidative stress in the fungal hyphae (Cao et al., 1992; Bassam et al., 2002). Mycelial development is slowed down, mycelium production is reduced, and spore germination is also inhibited by fungicidal volatiles (Chambers et al., 2013; Wu et al., 2015). They also interfere with gene expression and upregulate the transcription level of target genes related to redox reactions (glycerol-3-phosphate dehydrogenase, glutathione peroxidase, glutaredoxin, thioredoxin), cell wall integrity (chitin synthase, chitinase, and endo-1,3(4)-beta-glucanase) pathway, cell division control protein 6, programmed cell death protein 5, cell wall integrity, and stress response component (Baiome et al., 2022).

Except for *F. oxysporum*, the cell-free metabolites were broad-spectrum antifungal in nature and inhibited nine pathogens from various classes (Oomycetes, Phycomycetes, and Ascomycetes). CEL7 produced antifungal metabolites in its fermentation medium that were identified as phenol derivatives and imidazole compounds, which are well-known for their antibacterial qualities (Rani et al., 2013; Teodoro et al., 2015). The antifungal properties of *Alternaria tenuissima* PE2, an endophyte of *Psidium guajava*, were described by Chatterjee et al. in 2022. The current findings demonstrate that *C*. *elatior* endophytes synthesize new antifungals for agricultural use. The volatile metabolites of the endophytic fungus *Diaporthe* sp. CEL3, which suppresses *M*. *fructicola* infection in cherries, could be employed as a bio-control agent to stop infections during post-harvest and storage. Mycofumigation using CEL3 volatiles would control fungal infection, greatly reducing the need for chemical fungicides and potentially minimizing their harmful side effects. In turn, this would lead to the critically required shift to sustainable agricultural practices. Though *in vitro* conditions have allowed for the optimization of volatile emissions, improved carriers or media are still needed for random usage when transporting cherries or in storage facilities. Examples of low-cost agricultural waste items with high carbon-nitrogen ratios (C/N) that can be used to cultivate the fungi include bagasse, rice meal, wheat husk, wheat bran, tea seed pomace, sesame meal, and soybean meal. We are attempting to create a product that is appropriate for the growth of CEL3 with extended viability since these products still need to be optimized. Field-based validation is greatly encouraged for the more widespread use and commercialization of this endophyte.

## Conclusions

The camphor odor volatiles of the endophytic fungi *Diaporthe* sp. CEL3 exhibits antifungal action against pathogens such as *R*. *solani*, *B*. *cinerea*, and *P*. *ultimum* and causes 90% inhibition of fruit rot in *M*. *fructicola*-infected cherries. The major VOCs were geraniol, verbenol, and trans-ocimenol. CEL3 emits maximum VOCs in optimized media prepared using instantly smashed potato, yeast extract, dextrose, and wheat husk. This study illuminates that a volatile metabolite from endophytic fungi, *Diaporthe* sp. CEL3, an isolate of *C*. *elatior* leaf, could be used as a bio-control agent to minimize fungal infection-related crop loss in a sustainable agricultural approach. CEL3 could be used especially in managing post-harvest infections of cherries, solving the problem of *M*. *fructicola*-mediated economic damage in the cherry industry. Further field-based validation will be necessitated to establish the proper usage of this endophyte and its VOCs to control post-harvest fungal pathogens.

## Data availability statement

The datasets presented in this study can be found in online repositories. The names of the repository/repositories and accession number(s) can be found below: https://www.ncbi.nlm.nih.gov/genbank/, OQ190397, https://www.ncbi.nlm.nih.gov/genbank/, OQ195796.

## Author contributions

HS designed and performed the experiment, and prepared the manuscript. DB designed the experiment and finalized the manuscript. All authors contributed to the article and approved the submitted version.

## Supplementary material

Supplementary Figure 1 Light microscopic photographs of sterile hyphae of isolate **(A)**- CEL3 and **(B)**- CEL7 respectively.

Supplementary Figure 2
**(A)**- Antifungal activity of the emitted volatiles of endophytic fungi CEL3 against fungal pathogens **(A-J)**. **(B)**- Control PDA plates of the fungal pathogens grown without the interference of fungal VOCs.

Supplementary Figure 3
**(A)**- Cherry fruits infected with *M*. *fructicola*, b- Cherry fruits infected with *M*. *fructicola* and sprayed with fungicide.

Supplementary Figure 4
**(A)**- Antifungal activity of CEL7 extract against a– *M*. *fructicola*, **(B)**- *C*. *ulmi*, c– *R*. *solani*, d– *G*. *candidum*, e– *B*. *cinerea*, f– *C*. *beticola*, g– *F*. *oxysporum*, h– *P*. *ultimum*.

Supplementary Figure 5
**(A)**- Thin-layer chromatographic (TLC) analysis of EA fraction of CEL7, **(B)**- GC-MS chromatogram of the metabolites present in the Fraction C.

Supplementary Figure 6
**(A)**- GC-chromatogram of the VOCs synthesized by the endophytic CEL3.

Supplementary Figure 7 Chemical structures of volatile and non-volatile antifungal metabolites identified by GC–MS-NIST library.
