# Supplementary figures and images for: Antifungal activity of volatile and non-volatile metabolites of endophytes of Chloranthus elatior Sw
Source: Front Plant Sci. 2023 May 17;14:1156323. doi: 10.3389/fpls.2023.1156323 (PMC10229785; doi:10.3389/fpls.2023.1156323)

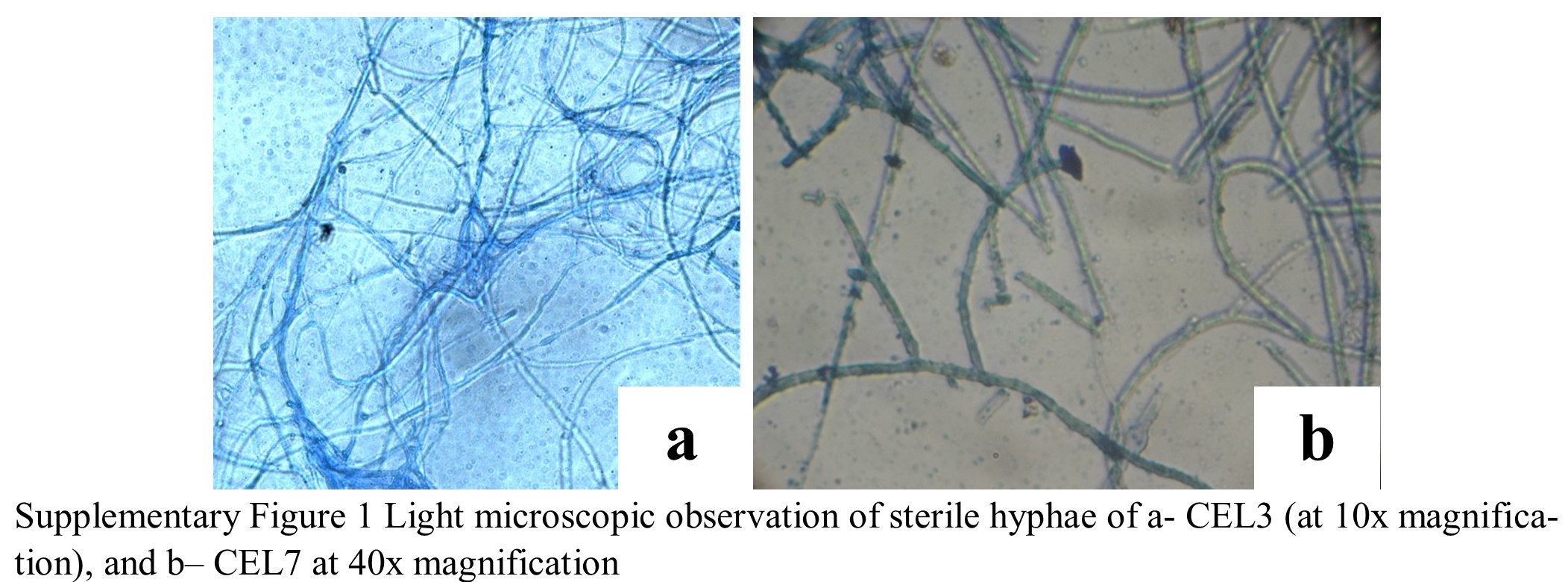

Supplement: Supplementary Figure 1 — Light microscopic photographs of sterile hyphae of isolate (A)- CEL3 and (B)- CEL7 respectively. [file Image_1.jpeg]

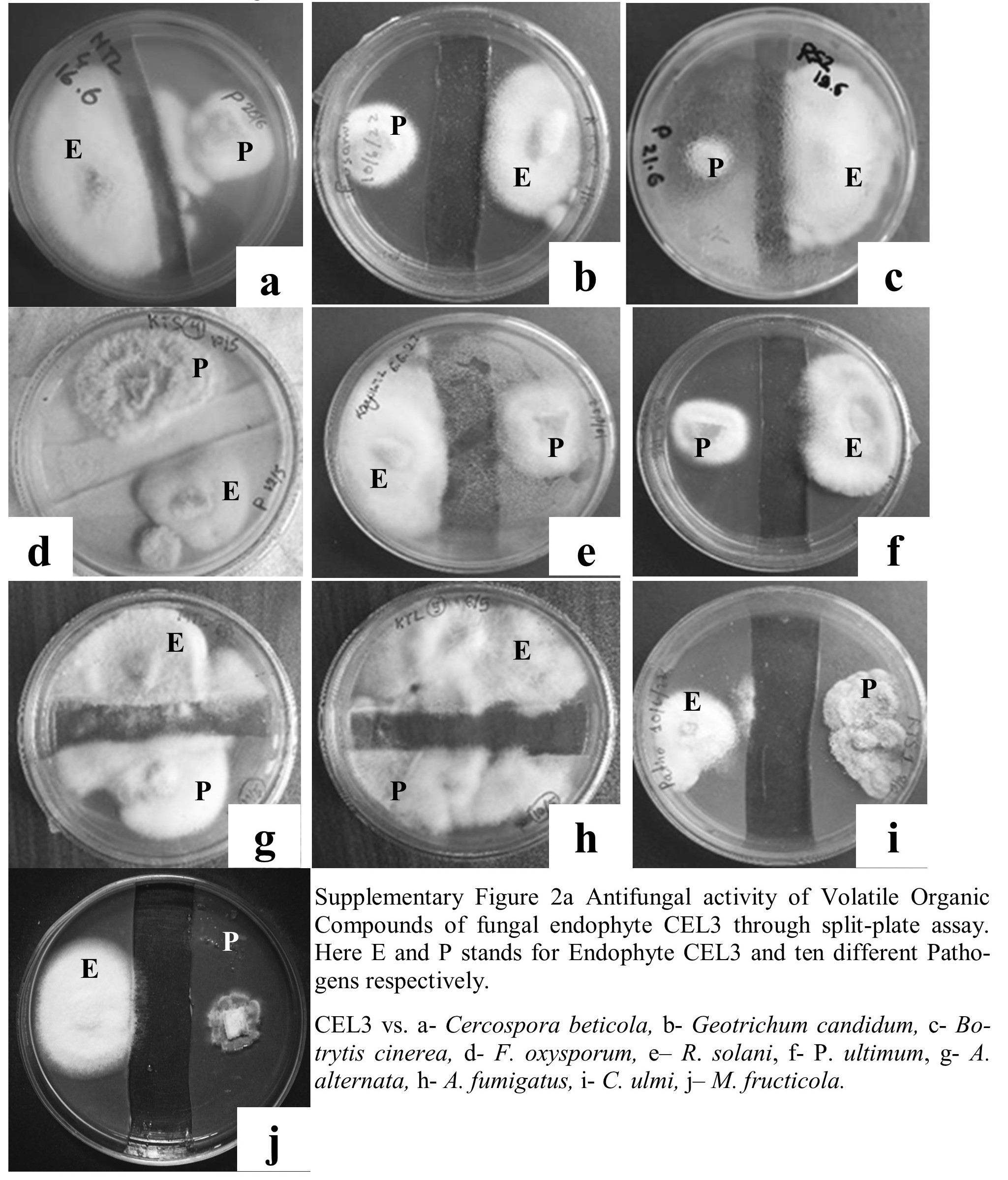

Supplement: Supplementary Figure 2 — (A)- Antifungal activity of the emitted volatiles of endophytic fungi CEL3 against fungal pathogens (A-J). (B)- Control PDA plates of the fungal pathogens grown without the interference of fungal VOCs. [file Image_2.jpeg]

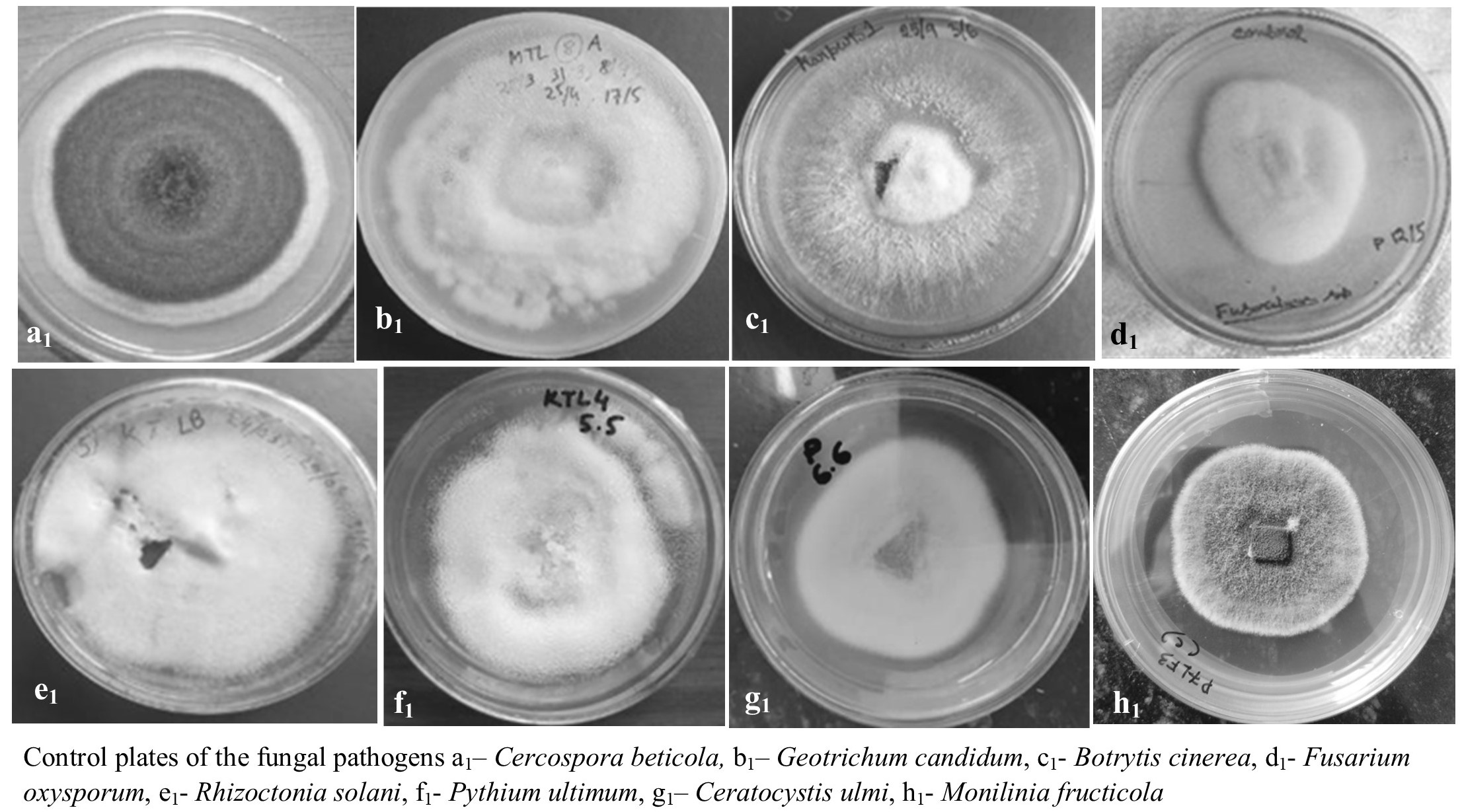

Supplement: Supplementary Figure 3 — (A)- Cherry fruits infected with M. fructicola, b- Cherry fruits infected with M. fructicola and sprayed with fungicide. [file Image_3.jpeg]

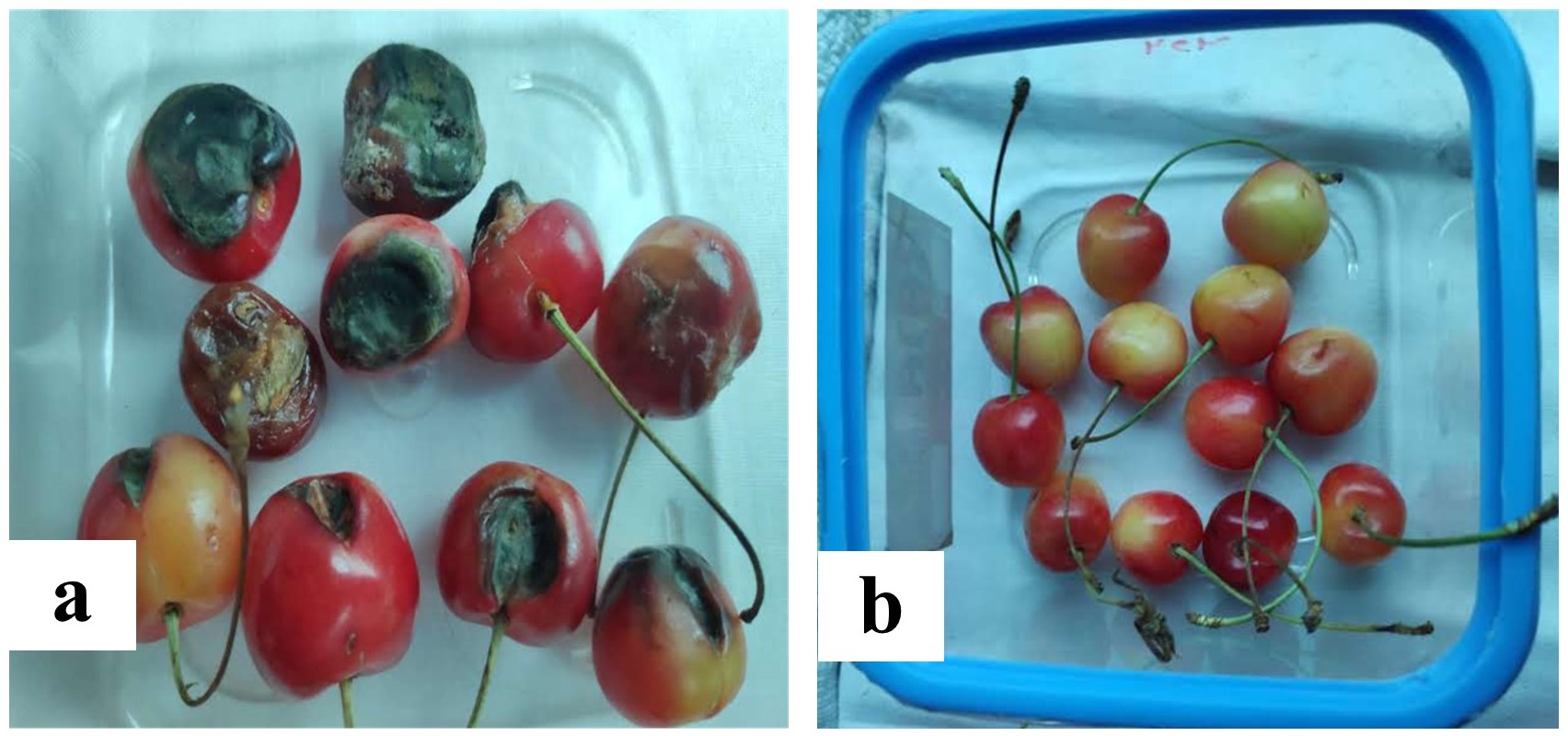

Supplement: Supplementary Figure 4 — (A)- Antifungal activity of CEL7 extract against a– M. fructicola, (B)- C. ulmi, c– R. solani, d– G. candidum, e– B. cinerea, f– C. beticola, g– F. oxysporum, h– P. ultimum. [file Image_4.jpeg]

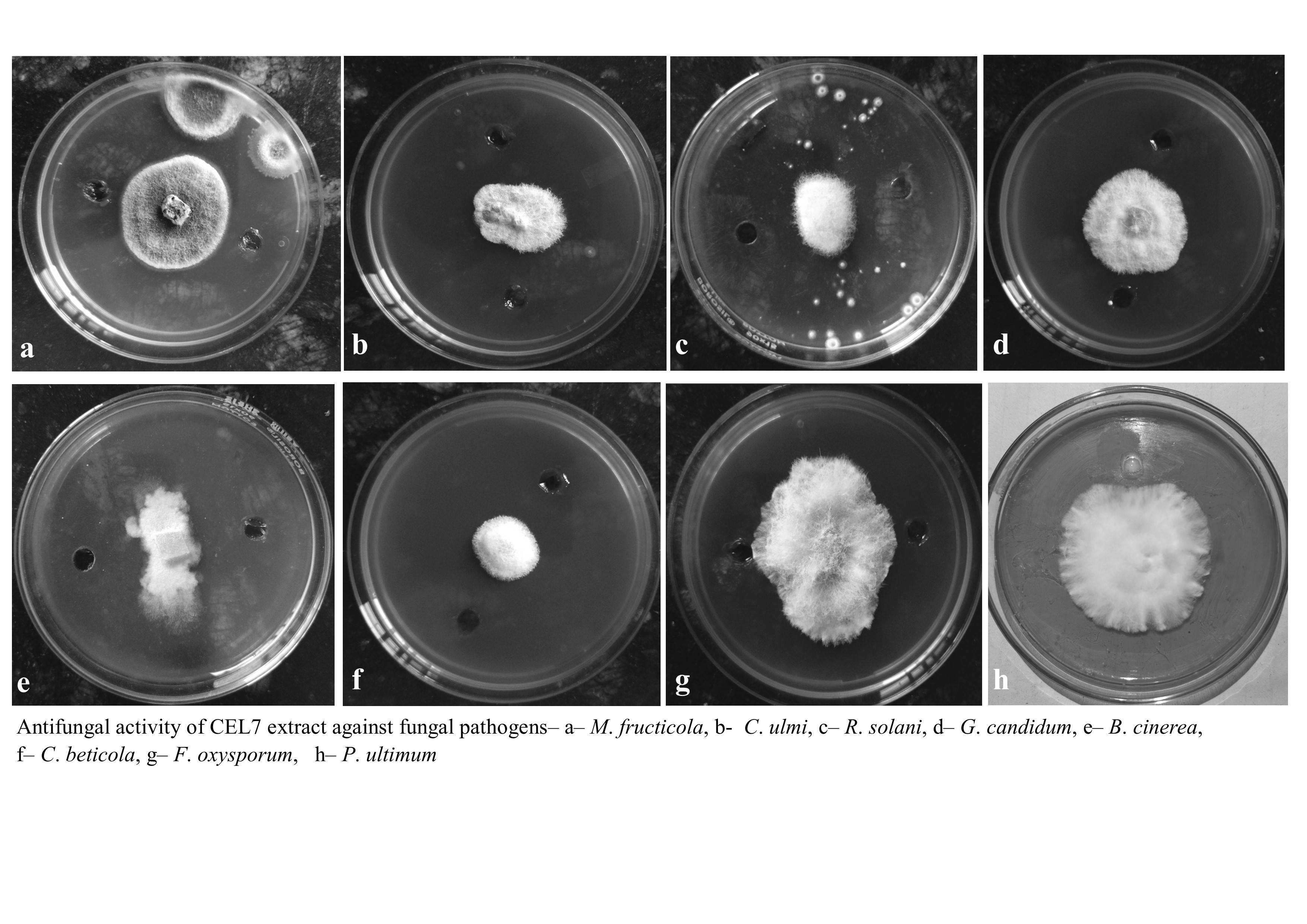

Supplement: Supplementary Figure 5 — (A)- Thin-layer chromatographic (TLC) analysis of EA fraction of CEL7, (B)- GC-MS chromatogram of the metabolites present in the Fraction C. [file Image_5.jpeg]

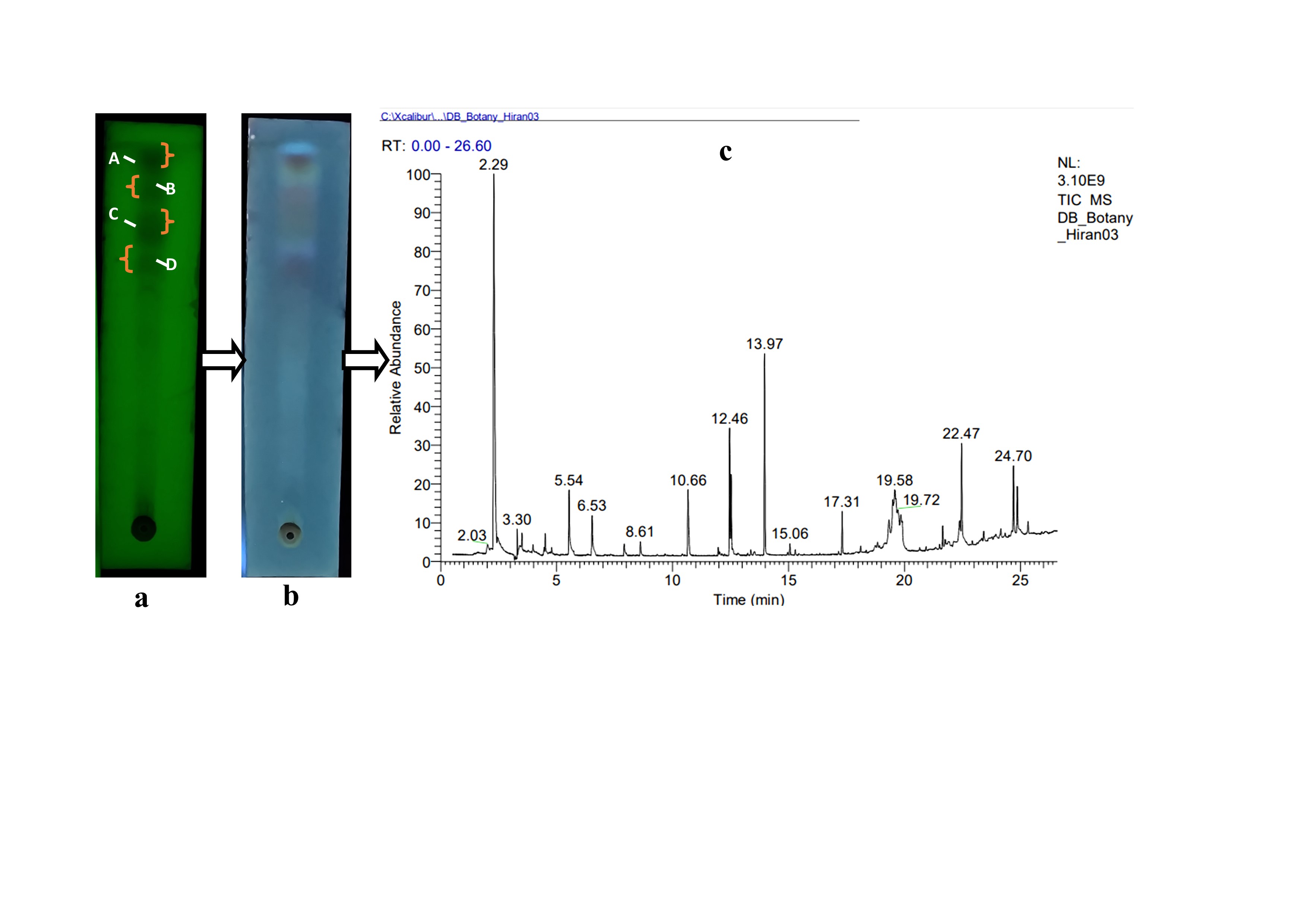

Supplement: Supplementary Figure 6 — (A)- GC-chromatogram of the VOCs synthesized by the endophytic CEL3. [file Image_6.jpeg]

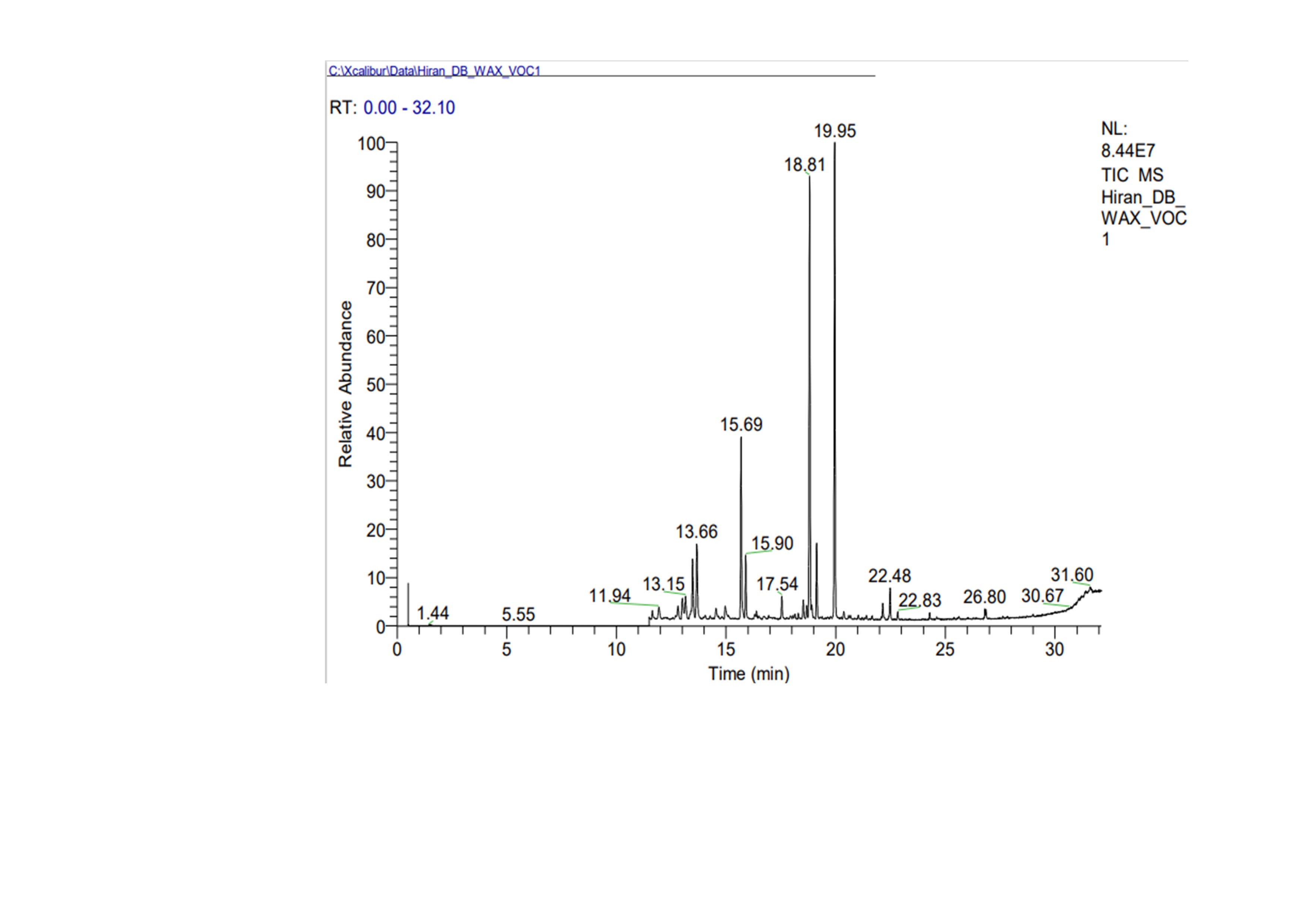

Supplement: Supplementary Figure 7 — Chemical structures of volatile and non-volatile antifungal metabolites identified by GC–MS-NIST library. [file Image_7.jpeg]

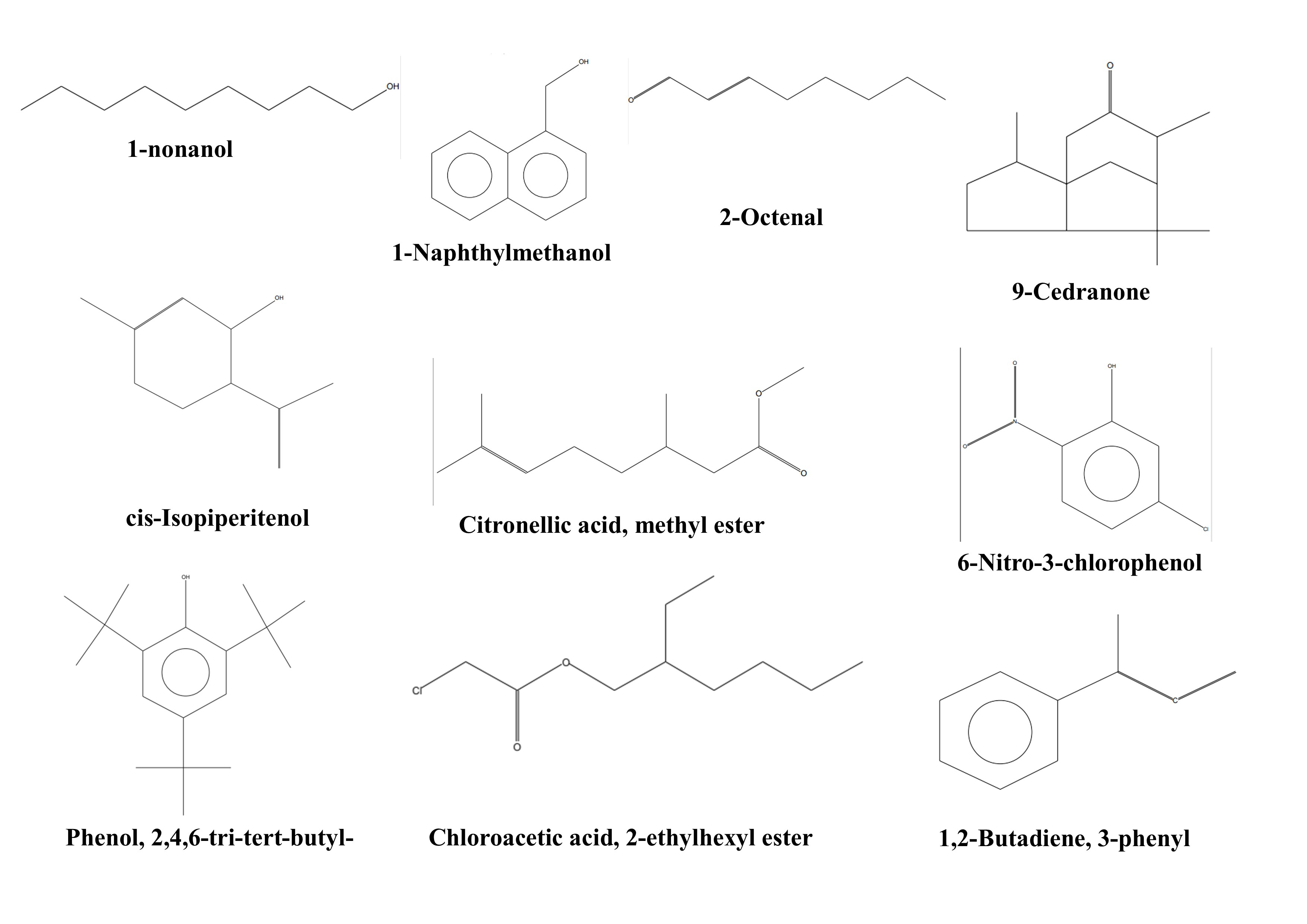

Supplement: Supplementary file 8 [file Image_8.jpeg]
